# siRNA Screen Identifies Trafficking Host Factors that Modulate Alphavirus Infection

**DOI:** 10.1371/journal.ppat.1005466

**Published:** 2016-03-31

**Authors:** Sheli R. Radoshitzky, Gianluca Pegoraro, Xiǎolì Chī, Lián Dǒng, Chih-Yuan Chiang, Lucas Jozwick, Jeremiah C. Clester, Christopher L. Cooper, Duane Courier, David P. Langan, Knashka Underwood, Kathleen A. Kuehl, Mei G. Sun, Yíngyún Caì, Shuǐqìng Yú, Robin Burk, Rouzbeh Zamani, Krishna Kota, Jens H. Kuhn, Sina Bavari

**Affiliations:** 1 Molecular and Translational Sciences Division, United States Army Medical Research Institute of Infectious Diseases (USAMRIID), Frederick, Maryland, United States of America; 2 Pathology Division, United States Army Medical Research Institute of Infectious Diseases (USAMRIID), Frederick, Maryland, United States of America; 3 Integrated Research Facility at Fort Detrick (IRF-Frederick), Division of Clinical Research (DCR), National Institute of Allergy and Infectious Diseases (NIAID), National Institutes of Health (NIH), Fort Detrick, Frederick, Maryland, United States of America; Purdue University, UNITED STATES

## Abstract

Little is known about the repertoire of cellular factors involved in the replication of pathogenic alphaviruses. To uncover molecular regulators of alphavirus infection, and to identify candidate drug targets, we performed a high-content imaging-based siRNA screen. We revealed an actin-remodeling pathway involving Rac1, PIP5K1- α, and Arp3, as essential for infection by pathogenic alphaviruses. Infection causes cellular actin rearrangements into large bundles of actin filaments termed actin foci. Actin foci are generated late in infection concomitantly with alphavirus envelope (E2) expression and are dependent on the activities of Rac1 and Arp3. E2 associates with actin in alphavirus-infected cells and co-localizes with Rac1–PIP5K1-α along actin filaments in the context of actin foci. Finally, Rac1, Arp3, and actin polymerization inhibitors interfere with E2 trafficking from the trans-Golgi network to the cell surface, suggesting a plausible model in which transport of E2 to the cell surface is mediated via Rac1- and Arp3-dependent actin remodeling.

## Author Summary

Alphaviruses, such as Chikungunya or Venezuelan equine encephalitis viruses, are significant human pathogens that cause arthritis or fatal encephalitis in humans. For productive infection of cells, alphaviruses rely on a repertoire of cellular host proteins, including trafficking factors that mediate transport of viral components across the cell. We have performed a functional screen to identify cellular factors that are crucial for this transport process. We show that Rac1, PIP5K1-alpha, and the Arp2/3 complex are cellular regulators of alphavirus infection. These factors are important for major cellular actin rearrangements that occur at a late stage of virus infection and are virus-induced. Concomitantly, these factors might be essential for trafficking of the viral E2 surface glycoprotein from the *trans-*Golgi network (TGN) to the cell surface. E2 was found to associate with actin, as well as to co-localize with Rac1, PIP5K1-α, and actin filaments. Late E2-containing vesicles, termed cytopathic vacuoles II (CPV-II), were also imaged along and at the end of actin filaments in alphavirus-infected cells.

## Introduction

Viral infection requires extensive subcellular trafficking, including cell entry, delivery of the genome to replication sites, and transport of viral proteins to and assembly of viral particles at the plasma membrane for egress. To this end, viruses make use of different cellular cues and signals to hijack existing endocytic and secretory pathways, cellular motor proteins, and cytoskeletal filaments.

Here we examine cellular trafficking machineries utilized by alphaviruses. Alphaviruses (family *Togaviridae*) are single-stranded, positive-sense RNA viruses that produce enveloped virions. Chikungunya virus (CHIKV), eastern equine encephalitis virus (EEEV), Venezuelan equine encephalitis virus (VEEV), and western equine encephalitis virus (WEEV) are the most medically important human alphaviruses that cause debilitating arthritides (CHIKV) or encephalitides (EEEV, VEEV, and WEEV) [1–3]. For instance, since December 2013, spread of CHIKV in the Caribbean has caused tens of thousands of human infections [4].

The alphavirus genome consists of two open reading frames encoding nonstructural and structural polyproteins. Four nonstructural proteins (nsP1-4) are required for transcription and replication of viral RNA, and three main structural proteins (i.e., capsid protein C, envelope glycoproteins E2 and E1) are the main constituents of virions. Alphavirus replication occurs initially at the plasma membrane [5,6]. Replication complexes are subsequently internalized via an endocytic process that requires a functional actin-myosin network. Following endocytosis, replication complex-containing vesicles migrate via a microtubule-dependent mechanism to the perinuclear area where they form stable, large and acidic compartments termed cytopathic vacuoles (CPV)-I. CPV-I structures are derived from modified endosomes and lysosomes and are associated with the alphaviral nonstructural proteins and viral RNA [7–9]. In the late stage of alphavirus infection, *trans Golgi* network (TGN)-derived vacuoles marked with the E1/E2 glycoproteins become predominant [10,11]. In these membrane vacuoles (termed CPV-II), the viral glycoproteins are arranged in a tubular structure. CPV-II vacuoles are implicated in intracellular transport of alphavirus glycoproteins from the TGN to the site of budding on the plasma membrane prior to virus egress [8,12].

Results from small interfering RNA (siRNA) screens identified a number of host factors that possibly promote or restrict nonpathogenic alphavirus infection [13–15]. However, detailed mechanistic studies regarding the role of host factors in alphavirus trafficking have not been performed. In this study, we used an RNAi-based screen to identify and validate trafficking host factors required for infection by the pathogenic VEEV and other pathogenic alphavirus relatives. Mutagenesis-, chemical inhibitor- and imaging-based approaches were further used to validate and decipher the role of these factors in alphavirus infection.

## Results

### High-Content RNAi Screen Identifies Host Trafficking Regulators of Alphavirus Infection

**Fig 1:**
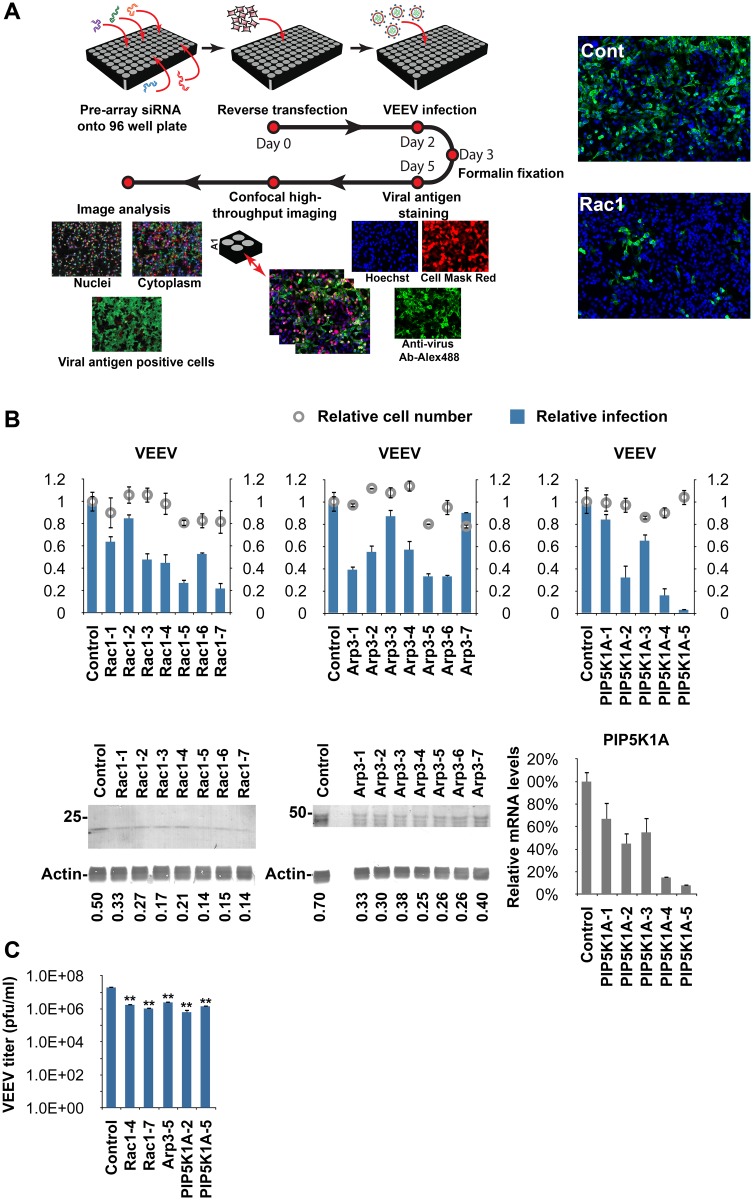
siRNA screen identifies host regulators of alphavirus infection. (**A**) Schematic representation of the siRNA screen and the high-content quantitative image-based analysis of relative VEEV infection rates. HeLa cells were transfected with siRNAs against 140 host trafficking factors and inoculated with VEEV (multiplicity of infection [MOI] = 0.5) for 20 h. Cells were fixed and immunostained for cell surface VEEV envelope glycoprotein (E2) expression. Infection rates were determined using an Opera confocal imager and normalized to infection rates observed using non-targeting control siRNA. Representative images of cells treated with control (Cont) or Rac1 siRNA are shown. VEEV E2 staining is shown in green, and nucleus staining is shown in blue. (**B**) High-content quantitative image-based analysis was used to measure relative infection rates (normalized to control siRNA-treated cells) of VEEV (top panel) in HeLa cells pretreated with the indicated siRNAs. Cells were infected for 20 h (VEEV, MOI = 0.5), fixed, and stained with antibodies against E2. Values represent the mean ± SD, n = 3. Protein levels of Rac1, Arp3, and actin (loading control) following siRNA treatments were determined by immunoblotting (bottom left 2 panels). mRNA levels of PIP5K1-α (PIP5K1A) following siRNA treatments were determined by quantitative reverse transcriptase polymerase chain reaction (qRT-PCR, bottom right panel). (**C**) VEEV titer following treatment of HeLa cells with siRNAs against Rac1, Arp3, PIP5K1A (PIP5K1-α), or control siRNA. Cells were inoculated with VEEV as in (**B**), and virus-containing media were analyzed by plaque assay. **, *p* <0.01, Student's *t* test (between the sample and control siRNA).

siRNA pools targeting each of 140 human trafficking genes were transfected into HeLa cells. A non-targeting siRNA was used as a control. Cells were subsequently infected with VEEV (chosen as a prototype alphavirus for the screen) for 20 h and then fixed and stained with a VEEV E2 glycoprotein-specific antibody (Fig 1A). Staining was performed without permeabilization to detect only E2 present on the cell surface. Cell number and infection rate were determined using quantitative high-content image-based analysis (see Materials and Methods). The infection rate of control siRNA-transfected cells was optimized to yield, on average, 70–80%. Analysis of the results revealed that siRNAs against 51 host trafficking factors decreased VEEV infection rate by >30% (Z-score <-2) (S1 Table).

To confirm results of the primary screen and to rule out potential off-target effects of individual siRNAs, we performed a secondary screen of deconvoluted siRNA pools. A hit was considered validated if at least 2 siRNAs from the set of 4 individual siRNAs targeting the gene product reduced the VEEV infection rate by ≥30% and had a *p*-value of <0.05 versus control siRNA-transfected wells. Wells that had low normalized cell numbers (final cell number <70% of the control siRNA-transfected well) due to combined effects of siRNA toxicity and VEEV cytopathic effects were excluded from further analyses. Analysis of the results led to validation of 19 (61%) out of the 31 primary hits (S2 Table).

Importantly, the list of validated hits was enriched for crucial regulators of the actin cytoskeleton. In particular, knockdown of four subunits of the heptameric Arp2/3 complex, ARPC4, ARPC5, ARPC1B (S2 Table), and ACTR3 (actin-related protein 3; Arp3) (Fig 1B), significantly inhibited VEEV infection. In addition, Ras-related C3 botulinum substrate 1 (Rac1), and phosphatidylinositol 4-phosphate 5-kinase type 1-alpha (PIP5K1-α or PIP5K1A) were also identified as hits (Fig 1B). The Arp2/3 complex plays a central role in actin dynamics by controlling filament nucleation [16,17]. Rac1 is a member of the Rho GTPase family and among its many functions modulates actin cytoskeleton organization [18]. PIP5K1-α is a lipid kinase involved in the synthesis of the signaling molecule phosphatidylinositol-4,5-bisphosphate (PI4,5P_2_), which is a central regulator of the actin cytoskeleton in response to multiple signals [19].

Our siRNA results were further confirmed using single siRNAs against Rac1, Arp3, and PIP5K1-α from another source (Fig 1B, siRNAs 5–7). We also observed a ≈10 to >30 fold reduction in VEEV titer following knockdown of these host factors (Fig 1C). Finally, siRNA-mediated knockdown of Rac1, Arp3, or PIP5K1-α inhibited infection of CHIKV (S1A and S1B Fig). These results indicate that Rac1, Arp3, and PIP5K1-α play an important role in alphavirus infections.

### Rac1 and Arp3 Inhibitors Reduce Alphavirus Infection Rates

**Fig 2:**
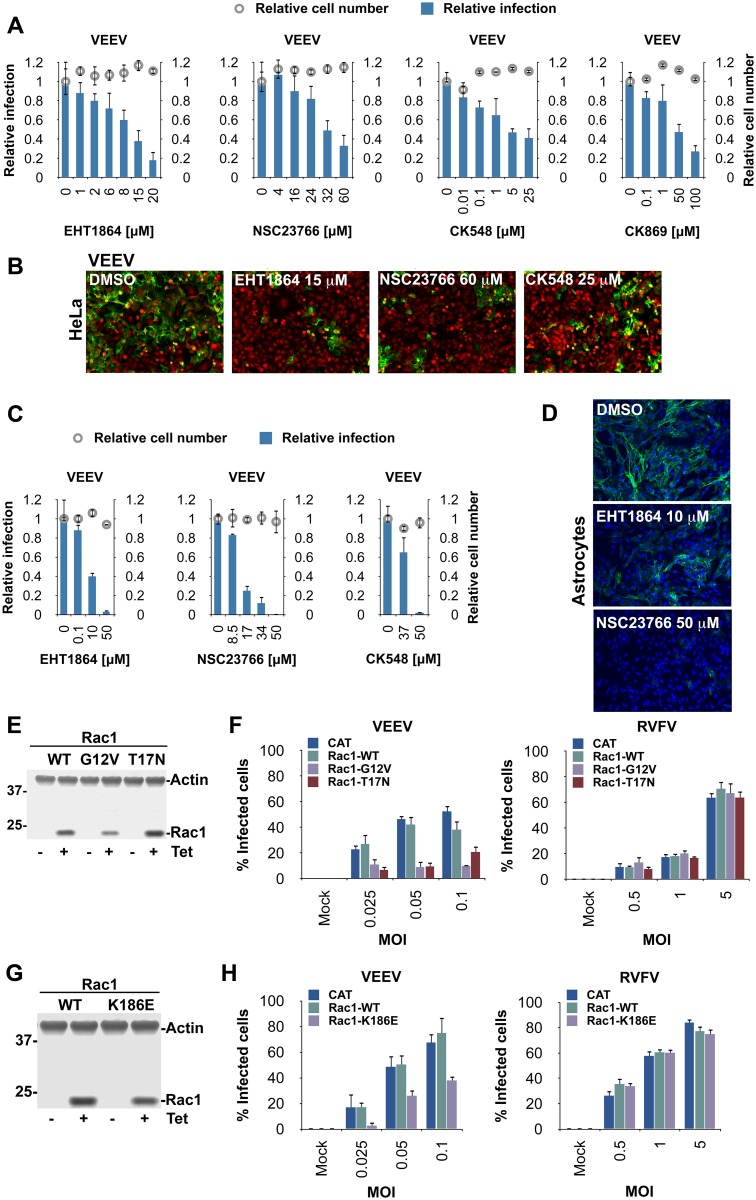
Rac1, Arp3 and formation of a Rac1:PIP5K1-α complex are important for VEEV infection. (**A**) High-content quantitative image-based analysis of relative VEEV infection rates in HeLa cells pretreated with increasing concentrations of two Rac1 inhibitors (EHT1864 or NSC23766), two Arp3 inhibitors (CK548 or CK869), or dimethyl sulfoxide (DMSO). Cells were inoculated with compounds 1 h prior to VEEV addition. Cells were fixed and stained with virus-specific antibodies 20 h later. Results are normalized to DMSO-treated samples. (**B**) Representative confocal images of (**A**). VEEV E2 staining is shown in green and nucleus/cytoplasm staining is shown in red. (**C**) Primary human astrocytes were treated with increasing concentrations of EHT1864, NSC23766, or CK548, and subsequently inoculated with VEEV (MOI = 0.005). Cells were fixed 19 h later, stained, and analyzed as in (**A**). (**D**) Representative confocal images of (**C**). VEEV E2 staining is shown in green and nucleus staining is shown in blue. (**E**) Flp-In T-REx 293 cells with tetracycline-inducible expression of wild-type Rac1, constitutively active Rac1 (G12V) or dominant-negative Rac1 (T17N) were generated, and analyzed for protein expression by immunoblotting (actin was used as a loading control). (**F**) High-content quantitative image-based analysis of VEEV or RVFV infection rates in Flp-In T-REx 293 cells pre-induced to express chloramphenicol acetyltransferase (CAT), wild-type Rac1, or variants thereof. Cells were fixed 18 h (VEEV) or 24 h (RVFV) after virus inoculation and stained with virus-specific antibodies. (**G**) Immunoblot of tetracycline-induced expression of wild-type Rac1, or Rac1 K186E in Flp-In T Rex 293 cells as in (**E**). (**H**) High-content quantitative image-based analysis of VEEV or RVFV infection rates in Flp-In T-REx 293 cells pre-induced to express CAT, wild-type Rac1, or Rac1 K186E. Cells were infected and stained as in (**F**).

To validate the role of Rac1 and Arp3 in VEEV infection, we tested whether the Rac1 inhibitors EHT1864 and NSC23766 [20,21] and the Arp3 inhibitors CK548 and CK869 [22] could block VEEV infection. Upon treatment of HeLa cells with either of these types of inhibitors, VEEV infection rates were reduced in a dose-dependent manner (Fig 2A and 2B). Similar results were observed when the Rac1 inhibitors EHT1864 or NSC23766 or the Arp3 inhibitor CK548, were tested in primary human astrocytes (Fig 2C and 2D). These inhibitors were also effective in reducing infection rates of other alphaviruses. EHT1864 inhibited infections by CHIKV and the closely related Sindbis virus (SINV), and CK548 decreased CHIKV, SINV, EEEV, and WEEV infection rates (S2A and S2B Fig). None of the treatment conditions in either assays resulted in cytotoxicity. Overall, our results further confirm the importance of host factors Rac1 and Arp3 in alphavirus infection.

### Rac1 GTPase Function and Rac1:PIP5K1-α Complex Formation Are Important for Alphavirus Infection

To determine if the function of Rac1 in alphavirus infection required Rac1’s GTPase activity, we established tetracycline-inducible 293 Flp-In T-REx cell lines that express chloramphenicol acetyltransferase (CAT, used as a control), wild-type Rac1, constitutively active Rac1 (G12V), or dominant-negative Rac1 (T17N) (Fig 2E) [23,24]. Rac1 expression in these cells was induced with tetracycline for 24 h, followed by infection with VEEV, or a non-alphavirus control (Rift Valley fever virus; RVFV strain ZH501, hereafter, RVFV). Expression of both Rac1 mutant variants (G12V, T17N) reduced VEEV but not RVFV infection rates, whereas expression of wild-type Rac1 had no effect (Fig 2F, S2C Fig). Both Rac1 mutants also reduced VEEV titer in the media (S2D Fig). We also confirmed the importance of Rac1 GTPase activity during WEEV and CHIKV infection (S2F and S2G Fig). The inhibitory effects of both Rac1 mutant variants on alphavirus infection likely indicate that the role of Rac1 during infection requires completion of the GTP-GDP-exchange/GTP-hydrolysis cycle. Cycling between GTP- and GDP-bound states may be required for productive infection, and shifting the level of activity predominantly to either side may block signaling pathways that emanate from the turnover.

Rac1 also forms a complex with PIP5K1 kinases that are necessary for stimulation of PI4,5P_2_ synthesis and actin assembly [25]. PIP5K1-α directly binds Rac1 via the polybasic tail of Rac1. Specific mutations within this region, such as K186E, abrogate Rac1:PIP5K1-α binding *in vitro* [26]. To examine whether Rac1:PIP5K1-α complex formation is important for VEEV infection, we used the tetracycline-inducible 293 Flp-In T-REx cell line to expresses Rac1 variant K186E (Fig 2G). Once induced, these cells and control cells expressing CAT or wild-type Rac1 were infected with VEEV or RVFV. Expression of Rac1 K186E reduced VEEV but not RVFV infection rates (Fig 2H, S2E Fig). VEEV titer in the media was also reduced (S2D Fig). Finally, we confirmed the importance of Rac1:PIP5K1-α complex formation to infection with CHIKV (S2H and S2I Fig). These results suggest that binding of Rac1 to PIP5K1-α plays a role in alphavirus infections.

### Rac1 and Arp3 Do Not Affect Alphavirus Cell Entry or Replication but Later Stages of Infection prior to Virus Budding

**Fig 3:**
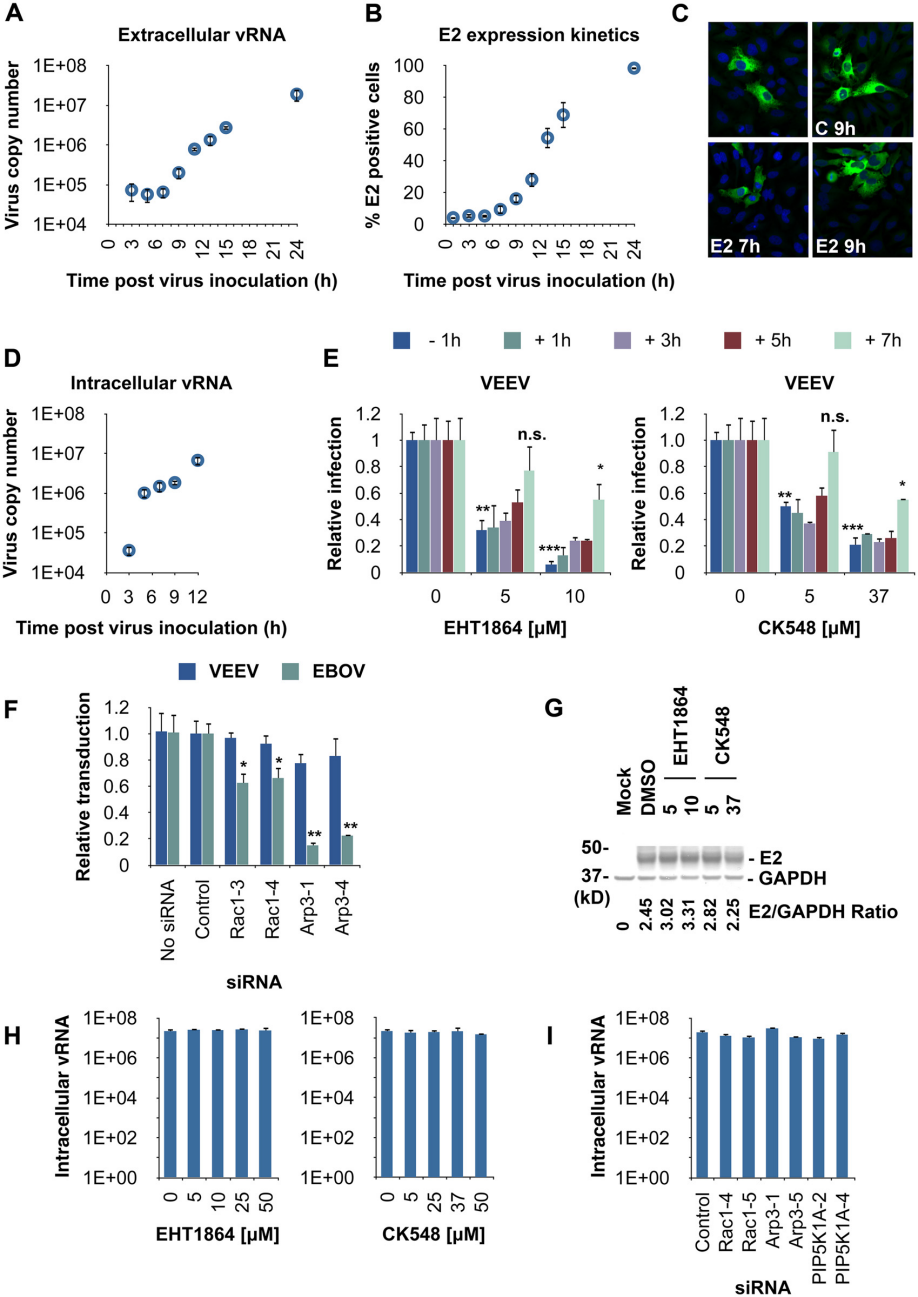
Rac1 and Arp3 act at a late stage of alphavirus infection. (**A** and **B**) Time course of VEEV TC-83 (MOI = 2) infection in HeLa cells. (**A**) Media containing extracellular virions were harvested at the indicated time points for qRT-PCR analysis of virus copy number, and (**B**) cells were fixed, stained with VEEV E2-specific antibody and analyzed with an Opera confocal reader by high-content quantitative image-based analysis. (**C**) Representative confocal images of E2 or C expression in VEEV-infected (MOI = 0.5) HeLa cells 7 h or 9 h following virus inoculation. Cells were stained with E2- or C-specific antibodies (green) and counterstained with dye to show the nuclei (blue). (**D**) Viral copy number (intracellular vRNA) in HeLa cells at the indicated time points following VEEV TC-83 (MOI = 10) inoculation was determined by qRT-PCR. (**E**) High-content quantitative image-based analysis of relative VEEV infection rates in time-of-addition experiments. VEEV-infected HeLa cells (MOI = 0.5) were treated with increasing concentrations of the inhibitors at the indicated time points prior to (-1 h) or after (+1–7 h) virus addition. Cells were fixed 20 h after addition of virus, stained and analyzed as in (**B**). Results are normalized to DMSO-treated samples. Values represent the mean ± SD, n = 3. *, *p* < 0.05; **, *p* < 0.01; ***, *p* < 0.001; n.s., not significant, Student's *t* test (between the sample and DMSO-treated cells). (**F**) HeLa cells were transfected with siRNAs targeting Rac1, Arp3, or control siRNA. Two days later, cells were transduced with enhanced green fluorescent protein (eGFP)-expressing MoMLV pseudotyped with the envelope proteins of VEEV (E1/E2) or Ebola virus (EBOV, GP_1,2_). eGFP-expressing cells were measured as in (**B**). Transduction rates were normalized to control siRNA-treated cells. *, *p* < 0.05; **, *p* < 0.01, Student's *t* test (between the sample and control siRNA) (**G**) Aliquots of the cells treated in (**E**) were lysed and analyzed for E2 expression by immunoblotting (GAPDH was used as a loading control). Densitometric analysis of western blots was performed with ImageJ. (**H-I**) VEEV copy number (intracellular vRNA) in HeLa cells following treatment with inhibitors or siRNAs as determined by qRT-PCR. (**H**) HeLa cells were inoculated with VEEV TC83 (MOI = 2) and treated 5 h later with the indicated inhibitors. Cells were lysed and analyzed for virus copy number 11 h after virus addition. (**I**) HeLa cells were treated with the indicated siRNAs and inoculated 48 h later with VEEV TC83 (MOI = 2). Cells were lysed and analyzed as in (**H**).

We used a multi-cycle VEEV in our screen. Consequently, Rac 1 and Arp3 could have acted at a number of stages of the VEEV lifecycle. To determine when Rac1 and Arp3 act, we first determined the time necessary for a single lifecycle (round) of VEEV TC-83 (live-attenuated vaccine strain) infection. We measured virus particle release from HeLa cells to the media at different time points post virus inoculation using qRT-PCR analysis. Virus particle release into the media was observed at 9 h post inoculation of HeLa cells (Fig 3A and S3A Fig, left panel), suggesting an approximately 9-h replication cycle for VEEV under these conditions. Expression kinetics of the late alphaviral gene product, E2, was also analyzed. E2 expression was detected as early as 7 h post virus inoculation (Fig 3B and S3A Fig, right panel). Experiments performed with virulent VEEV IC-SH3 yielded similar results on expression of E2 and C proteins at these time points (Fig 3C). We confirmed our results with a one step-like growth curve analysis using a high MOI (MOI = 10) and also measured intracellular viral RNA (vRNA) levels as a function of time. Significant increase in intracellular vRNA levels was found at 5 h post virus inoculation, suggesting that virus replication/transcription is initiated prior to this time point (Fig 3D).

To narrow down the lifecycle stage targeted by Rac1 and Arp3, we performed time-of-addition experiments using inhibitors of these host factors. This time-based approach determines how long the addition of a compound can be postponed before losing its antiviral activity in cell culture. For example, if an inhibitor that targets viral fusion is present at the time when virus entry and fusion occurs within the viral lifecycle, productive infection will be inhibited. In contrast, if this inhibitor is added after the entry/fusion process is completed, the inhibitor will no longer be effective in blocking infection.

As a positive control for infection inhibition, HeLa cells were pretreated with increasing concentrations of Rac1 or Arp3 inhibitors 1 h before addition of virus. Alternatively, inhibitors were added to the cells at different time points after virus inoculation (1, 3, 5, or 7 h, Fig 3E) but prior to virus release (9 h post inoculation). When the Rac1 inhibitor EHT1864 or the Arp3 inhibitor CK548 were added 1, 3, or 5 h after VEEV exposure, VEEV infection rates were reduced to that detected with the positive control condition (pretreatment). However, addition of inhibitors 7 h after virus inoculation had significantly less effect on infection, suggesting that the inhibitors lose their antiviral activity at this time. Similar results were obtained with VEEV TC-83 in the context of a single replication cycle (S3B Fig); both EHT1864 and CK548 inhibitors reduced VEEV TC-83 infection when they were added up to 7 h post inoculation. Furthermore, when the inhibitors were added to HeLa cells 5 h following VEEV inoculation, VEEV titer in the media was significantly reduced (approximately 80- to >7,000-fold reduction, S3C Fig). Since the inhibitors exhibited antiviral activity when they were added 5 h post virus inoculation but significantly lost their antiviral affect when they were added 7 h post virus inoculation, these results indicate that Rac1 and Arp3 most likely play a role in the VEEV life cycle sometime between 5 h and 7 h post virus inoculation. Since one lifecycle of the virus takes at least 9 h to complete, and since transcription/replication is initiated prior to 5 h post virus inoculation, these results indicate that these inhibitors act at a late stage of virus infection.

To further confirm that Rac1 and Arp3 do not act at earlier stages (entry and replication), we first utilized a VEEV cell entry surrogate system composed of retroviral pseudotypes (Moloney murine leukemia virus; MoMLV) encoding eGFP and carrying the viral envelope proteins [27,28]. HeLa cells pretreated with control siRNA or with siRNAs targeting Rac1 or Arp3 were transduced with MoMLV-VEEV or MoMLV-EBOV (non-alphavirus control). As previously reported, MoMLV-EBOV entry into HeLa cells was reduced following knockdown of Rac1 or Arp3 [29,30] (Fig 3F). However, Rac1 or Arp3 knockdown had no or minimal effect on MoMLV-VEEV transduction rates, indicating that envelope-mediated entry of VEEV is independent of these two proteins.

Next, we examined the effect of the various inhibitors on total E2 protein levels in the context of virus infection. None of the inhibitors had an effect on E2 protein levels as determined by western blot analysis (Fig 3G).

Finally, we tested the effect of Rac1 and Arp3 on alphavirus replication in infected cells by treating cells with siRNAs as described above or with inhibitors against Rac1 or Arp3. Intracellular vRNA copy numbers were determined by qRT-PCR. The siRNAs as well as the inhibitors had no significant effect on intracellular vRNA copy numbers (Fig 3H and 3I). Similar results were obtained when the inhibitors were tested for their effect on CHIKV replication using a previously published replicon system (S3D Fig [31]). Overall, these results indicate that Rac1 and Arp3 function after virus entry and replication, but prior to budding and release.

### Actin Plays a Dual Role in Alphavirus Infection

As mentioned above, Rac1, Arp3, and PIP5K1A all affect cellular actin dynamics [16–19]. Previous studies have demonstrated a role for actin in alphavirus infection [32,33]. For example, in the early stages of infection of another alphavirus, Semliki Forest virus, replication complexes are internalized via an endocytic process that requires a functional actin-myosin network [7]. However, our time-of-addition experiments suggest that Rac1 and Arp3 play a role later in infection. We therefore investigated whether actin dynamics might play an additional role at later stages of infection.

**Fig 4:**
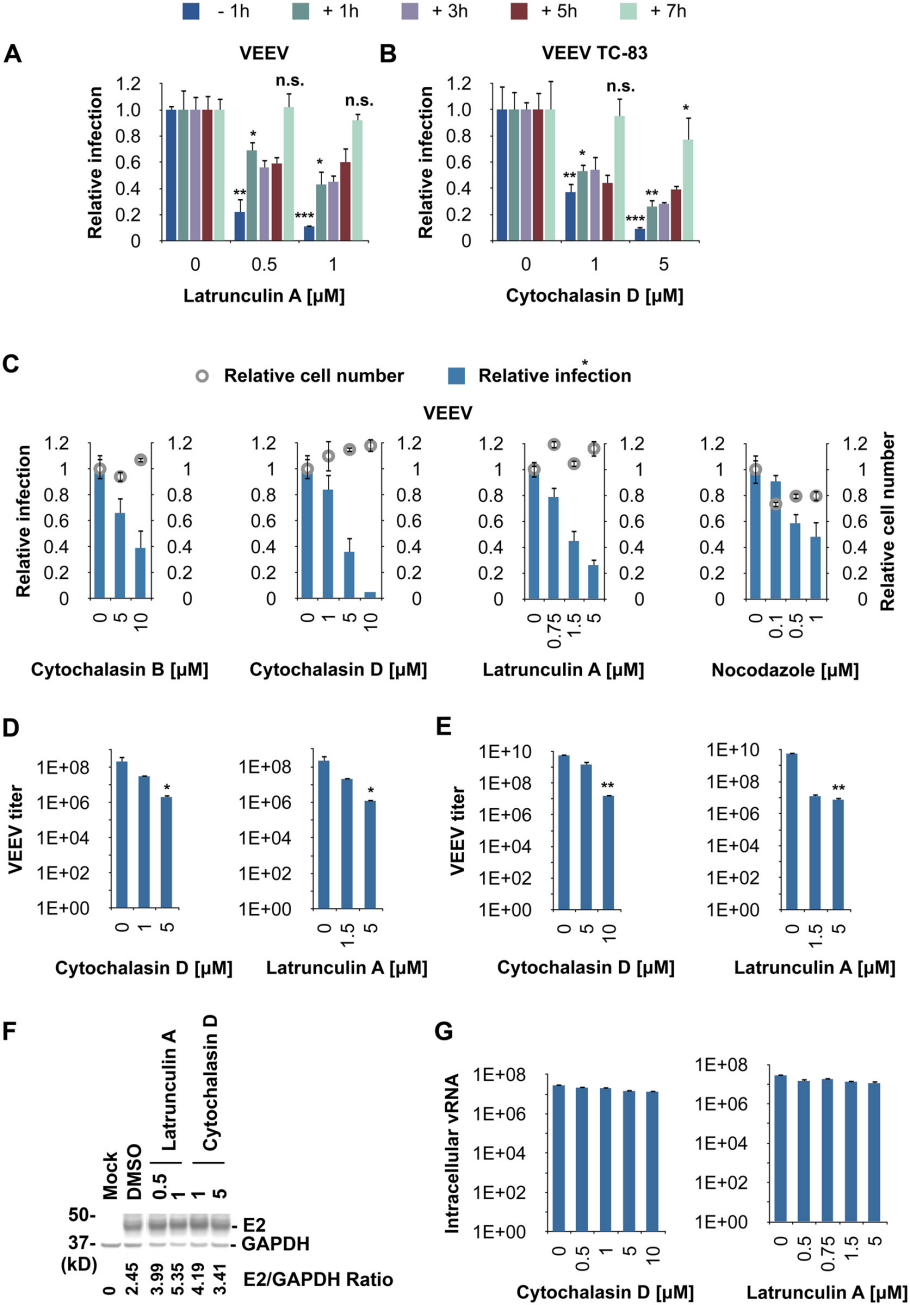
Actin polymerization plays a role at a late stage of alphavirus infection. (**A** and **B**) High-content quantitative image-based analysis of relative VEEV and VEEV TC-83 infection rates in time-of-addition experiments. (**A**) VEEV-infected HeLa cells (MOI = 0.5) were treated with increasing concentrations of latrunculin A at the indicated time points prior to (-1 h) or after (+1–7 h) virus addition. Cells were fixed 20 h after addition of virus and stained for high-content quantitative image-based analysis with virus-specific antibodies. (**B**) VEEV TC-83 (MOI = 1)-infected HeLa cells were treated with cytochalasin D as in (**A**). Cells were fixed 12 h after addition of virus, stained, and analyzed as in (**A**). (**C**) HeLa cells were infected with VEEV (MOI = 0.5) for 3 h and then treated with increasing concentrations of cytochalasin B, cytochalasin D, latrunculin A, or nocodazole. Cells were fixed in formalin 17 h later, stained, and analyzed as in (**A**). (**A-C**) Results are normalized to DMSO-treated samples. (**D**) HeLa cells were infected as in (**C**) for 3 h and then treated with increasing concentrations of cytochalasin D or latrunculin A. After 17 h, virus titer in the supernatants was determined by plaque assay. Values represent the mean ± SD, n = 2. (**E**) Primary human astrocytes were infected with VEEV TC-83 (MOI = 0.005) for 5 h and then treated with increasing concentrations of inhibitors. After 6 h, virus titer in the supernatants was determined by plaque assay. (**F**) Aliquots of the cells treated in (**A**) were lysed and analyzed for E2 expression by immunoblotting (GAPDH was used as a loading control). Densitometric analysis of western blots was performed with ImageJ. (**G**) VEEV copy number (intracellular vRNA) in HeLa cells following treatment with inhibitors was determined by qRT-PCR. HeLa cells were inoculated with VEEV TC-83 (MOI = 2) and 5 h later treated with the indicated inhibitors. Cells were lysed and analyzed for virus copy number 11 h after virus addition. (**A-C, E, G**) Values represent the mean ± SD, n = 3. *, *p* < 0.05; **, *p* < 0.01; ***, *p* < 0.001; n.s., not significant, Student's *t* test (between the sample and DMSO-treated cells).

To this end, we performed time-of-addition experiments (similar to the ones described above) with actin polymerization inhibitors. Cells were either pretreated with increasing concentrations of inhibitors before addition of virus (positive control) or preincubated with virus and subsequently treated with inhibitors at different time points after infection (Fig 4A and 4B).

Compared to the positive control condition (pretreatment), the actin polymerization inhibitors, latrunculin A and cytochalasin D, were less effective in inhibiting VEEV infection when they were added 1 h after virus inoculation (Fig 4A and 4B). This loss of antiviral activity is possibly due to the previously described role of actin in internalization of alphavirus replication complexes [7]. Inhibition of VEEV infection rates remained similar if actin polymerization inhibitors were added up to 5 h after virus inoculation. However, additional loss of antiviral activity was observed when the inhibitors were added at 7 h post virus inoculation. These results suggest that actin polymerization inhibitors target two separate steps in VEEV’s life cycle, one early in infection and one late in infection.

To further validate our results that actin might play a role in the later stages of the alphavirus lifecycle, we tested the effect of various doses of actin polymerization inhibitors (latrunculin A, cytochalasin B and D) or a microtubule-depolymerizing agent (nocodazole) on VEEV infection rate when added at various time points post virus inoculation. HeLa cells and primary human astrocytes were inoculated with VEEV first, and inhibitors were added 3 (HeLa) or 5 (astrocytes) h later. Disruption of actin dynamics by the actin polymerization inhibitors reduced VEEV infection rates and VEEV titer in a dose-dependent manner without cytotoxicity (Fig 4C–4E). Although some nocodazole-mediated inhibition of viral infection was observed, inhibition was not as marked as that observed with actin polymerization inhibitors and was accompanied by increased cytotoxicity (Fig 4C and S4A Fig). Phalloidin and tubulin staining demonstrated that the actin and microtubule cytoskeleton morphology was indeed disrupted upon treatment with these inhibitors (S4B and S4C Fig). These results further imply that actin polymerization might have an essential role in later stages of VEEV infection.

To determine if the actin polymerization inhibitors (latrunculin A and cytochalasin D) might block viral replication or E2 expression at later stages of infection, we inoculated cells with VEEV TC83 and treated them 5 h later with the inhibitors. Intracellular vRNA copy numbers were determined by qRT-PCR 11 h after virus inoculation. Alternatively, cells were lysed and analyzed for E2 expression by immunoblotting. Both inhibitors had no significant effect on vRNA copy numbers and E2 expression levels (Fig 4F and 4G). Finally, no effect on virus replication was observed when the actin polymerization inhibitors were tested for their effect on a CHIKV replicon system (S4D Fig) [31]. Together, the data suggests that the role of actin in the later stages of infection does not involve viral replication or late gene expression.

### Alphavirus Infections Cause Major Intracellular Actin Rearrangements Late in Infection

**Fig 5:**
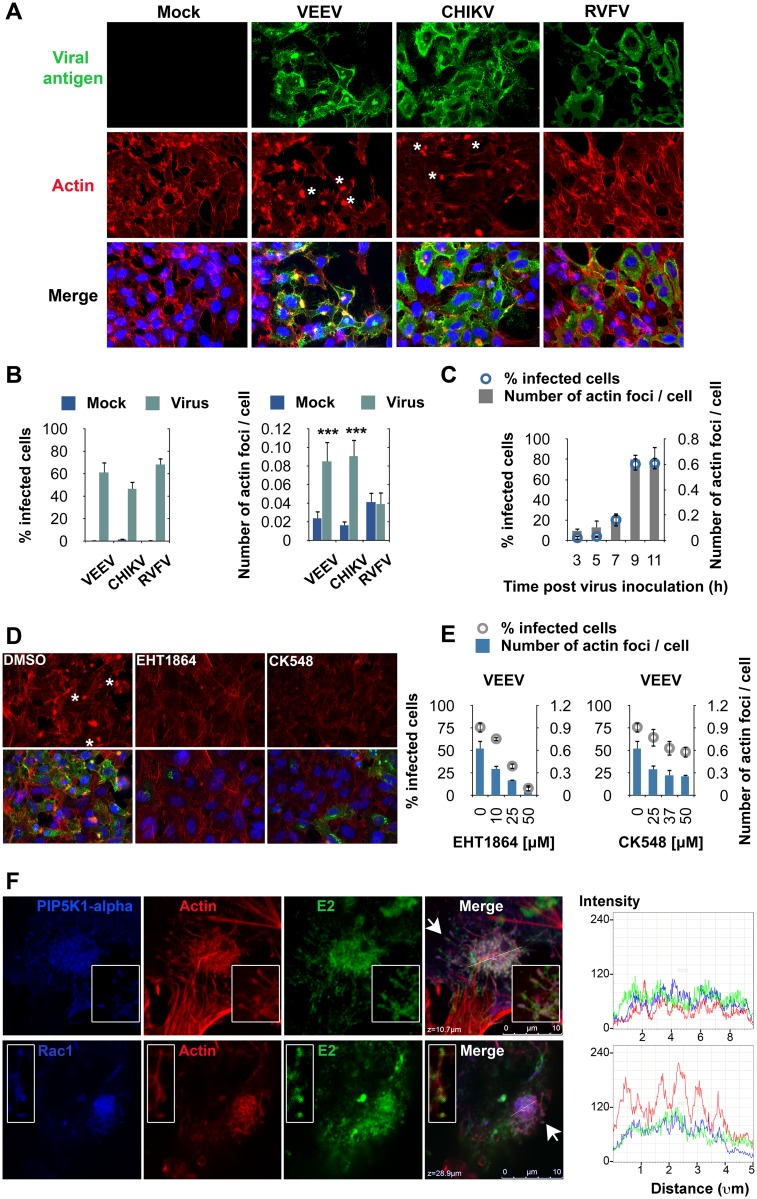
Alphavirus infection causes actin rearrangements into actin foci that are Rac1- and Arp3-dependent and that co-localize with Rac1, PIP5K1-α, and E2. (**A**) Representative confocal images of mock-, VEEV-, CHIKV-, or RVFV-infected HeLa cells (MOIs = 0.5, 5, or 3, respectively). Cells were fixed and stained with virus-specific antibodies (VEEV and CHIKV E2, RVFV nucleoprotein; shown in green) and phalloidin (red) 18 h (VEEV) or 24 h (CHIKV, RVFV) after infection. Nucleus staining is shown in blue. Representative actin foci are indicated by asterisks. (**B**) High-content quantitative image-based analysis was used to measure infection rates of VEEV, CHIKV, and RVFV (left panel), and the number of actin foci per cell (number of actin foci/total cell number, right panel). Analysis is based on single Z sections. ***, *p* < 0.0001, Student's *t* test (between the sample and mock). (**C**) VEEV-infected HeLa cells (MOI = 0.5) were fixed in formalin at the indicated time points, stained, and analyzed as in (**B**). (**B**–**C**) Values represent the mean ± SD, n ≥12. (**D**) Representative confocal images of VEEV-infected HeLa cells (MOI = 0.5) pretreated with the Rac1 inhibitor EHT1864 or Arp3 inhibitor CK548. Cells were fixed 18 h after virus addition and stained with VEEV E2-specific antibody (green), phalloidin (red), and a nuclear stain (blue). (**E**) High-content quantitative image-based analysis was used to measure infection rates of VEEV and the number of actin foci per cell. (**F**) Confocal images of VEEV-infected HeLa cells (MOI = 5). Co-localization of hemagglutinin (HA)-tagged PIP5K1-α (top panel) or Rac1 (bottom panel) (blue), actin (red), and VEEV E2 (green), at a single z section is shown (left panel). Insets: zoom on actin filaments indicated by white arrows. Single channel intensities were measured along lines crossing different actin clusters (right panel). VEEV was added to HeLa cells that were reverse-transfected with a plasmid encoding HA-tagged PIP5K1-α or tetracycline-induced T-Rex HeLa cells that expressed Rac1 fused to eGFP. Cells were fixed 20 h later, permeabilized, and stained with VEEV E2-specific antibody, phalloidin, and an antibody against HA.

To assess the possible role of actin in the later stages of alphavirus infection, we assessed temporal changes of actin rearrangements during the course of viral infection. HeLa cells were infected with VEEV, CHIKV, or RVFV (used as a control) and co-stained at the indicated time points with antibodies against viral proteins and phalloidin. Confocal microscopy revealed major changes in the actin-staining pattern within alphavirus-infected cells (VEEV, CHIKV), as indicated by the accumulation of actin in large structures in the cytoplasm (i.e., actin foci, indicated by asterisks in Fig 5A). These foci co-localized with the alphavirus envelope protein E2 (Fig 5A). In contrast, such actin rearrangements were not observed in RVFV- or mock-infected cells (Fig 5A).

Actin foci were further quantified (measured as the number of foci per cell) in mock-, VEEV-, CHIKV-, and RVFV-infected cells (Fig 5B). These foci were detected as early as 7 h after VEEV inoculation (Fig 5C) and could also be detected upon infection with other alphaviruses (EEEV, WEEV, and SINV, S5A Fig). We also tested whether alphavirus nsP1, which was previously shown to mediate disruption of actin stress fibers and induction of filopodia-like extensions [34], could induce generation of actin foci. Expression of VEEV TC83 nsP1 in HeLa cells did induce filopodia-like extensions. However, no actin foci were observed (S5B Fig). Overall, our results demonstrate that, as early as 7 h post inoculation with alphaviruses, infection causes major cellular actin rearrangements leading to the formation of actin foci that are not nsP1-dependent and that co-localize with the alphavirus envelope protein E2.

### Rac1 and Arp3 Inhibitors Reduce Actin Focus Formation in Alphavirus-Infected Cells

Because our data suggested that the timing of the effects of Rac1 and Arp3 and the formation of actin foci take place late in infection (Figs 3 and 5), we speculated that Rac1 and Arp3 proteins might play a role in this alphavirus-induced actin remodeling. To test this hypothesis, HeLa cells were treated with increasing concentrations of Rac1 or Arp3 inhibitors, infected with VEEV, and subsequently stained with fluorescent phalloidin and antibodies against E2. Treatment with either the Rac1 (EHT1864) or Arp3 (CK548) inhibitor significantly reduced the number of actin foci and the percentage of infected cells in a dose-dependent manner (Fig 5D and 5E). In fact, under these conditions actin foci were rarely observed in confocal images even in E2-positive cells. These observations clarify that Rac1 and Arp3 function upstream of the major actin rearrangements detected in VEEV-infected cells.

### Rac1 and PIP5K1-α Co-localize with E2 on Actin Foci and Actin Filaments

Since Rac1-PIP5K1-α complex formation plays a role in alphavirus infection (Fig 2) and because Rac1 inhibitor reduced actin foci formation in alphavirus-infected cells (Fig 5), we next examined whether both host factors could be observed on actin foci and/or filaments within alphavirus-infected cells. Basal-to-apical confocal section series of VEEV-infected HeLa cells are shown in Fig 5F. PIP5K1-α and Rac1 show increased co-localization with actin foci and E2 towards the apical area (S5C and S5D Fig). Both host factors are also detected along actin filaments, where they co-localize with E2 (Fig 5F, insets).

### Organization and Morphology of Actin Foci and Their Co-localization with the E2 Glycoprotein

**Fig 6:**
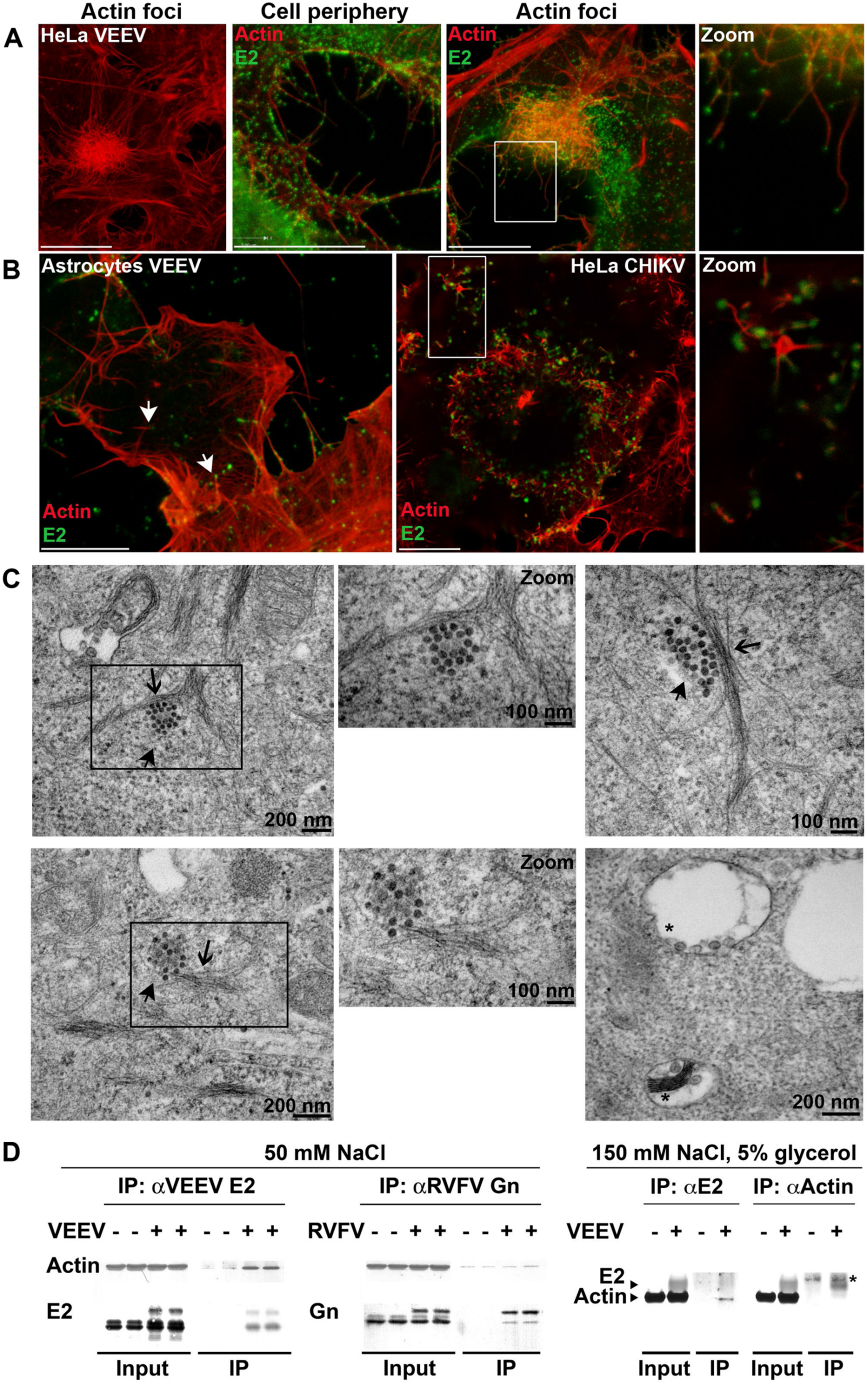
Alphavirus E2 co-localizes with actin filaments and associates with actin. (**A-B**) Representative STED images of HeLa cells or primary human astrocytes infected with VEEV or with CHIKV (MOI = 5). Cells were fixed, permeabilized, and stained with E2-specific antibodies (green) and phalloidin (red). Scale bar: 10 μm. (**C**) Electron-microscopic images of VEEV-infected HeLa cells (MOI = 5). CPV-II structures and thin filaments, which probably correspond to actin, are indicated by filled and open arrows, respectively. An asterisk indicates CPV-I structures. (**D**) Western blot analysis of input lysates and immunoprecipitates (IP) of mock-, VEEV-, or RVFV-infected HeLa cells under different lysis conditions. Cells were infected for 8 h (MOI = 1), lysed, and VEEV E2-, RVFV Gn-, or actin-binding proteins were immunoprecipitated using specific antibodies and immunoblotted with antibodies against VEEV E2, RVFV Gn, or actin. (*) indicates a non-specific band.

To better characterize the nature of the observed actin foci within infected cells, we performed sequential scanning of cells stained for actin and alphavirus E2 in both stimulated emission depletion (STED) microscopy and confocal microscopy imaging modes (for comparison, see S6A Fig). With improved resolution of STED microscopy, actin foci within infected cells were found to be clusters of filamentous actin with a diameter range of 5–11 μm (Fig 6A). Actin filaments within the clusters are seen with VEEV E2 puncta at their ends or along them (Fig 6A). On the cell periphery, E2 puncta are localized in proximity to actin filaments (Fig 6A). E2 puncta are also observed at the ends of actin filaments in primary human astrocytes, and in CHIKV-infected HeLa cells (Fig 6B).

In a series of basal- (Section 7) to-apical (Section 25) confocal sections, a single VEEV-infected cell can be seen with an actin cluster (S6B Fig). E2 co-localizes with the actin cluster, and cytoplasm/nucleus staining demonstrates that the generated actin cluster is localized within the cell (S6C Fig). In contrast, co-localization of E2 and microtubules was not significant (S6B Fig).

We also performed electron microscopic studies to examine the localization of cytoskeletal elements relative to alphaviral CPV-II structures. These structures compartmentalize the viral glycoproteins E1 and E2 and serve as transport vehicles for the glycoproteins from the TGN to the viral budding sites on the plasma membrane. Electron-microscopic studies of VEEV-infected cells (Fig 6C) show CPV-II structures alongside or at the end of thin filaments, which, based on size and morphology, most likely correspond to actin filaments [12]. CPV-I replication compartments are also present within these cells (Fig 6C, bottom right panel) [9].

### Alphavirus E2 Glycoprotein Associates with Actin

Because alphavirus E2 co-localized with actin filaments in infected cells, we next tested whether VEEV E2 associates with actin. HeLa cells were infected with VEEV or RVFV (control) or left uninfected (mock). Virus envelope protein-binding factors were subsequently immunoprecipitated from cell lysates with antibodies to surface glycoproteins E2 (VEEV) or Gn (RVFV). Western blot analysis of the immunoprecipitated fraction (IP) showed enrichment of actin in E2 immunoprecipitates from VEEV-infected cells relative to mock-infected control (more than 4-fold increase by densitometry analysis, Fig 6D, left panel). Such an increase in immunoprecipitated actin was not observed or was minimal in Gn immunoprecipitates from RVFV-infected cells (1.5-fold or less increase by densitometry analysis, Fig 6D, middle panel). To confirm the E2-actin association, we repeated these immunoprecipitation assays using more stringent lysis and washing conditions and performed the reverse experiment using an antibody against actin to examine its ability to immunoprecipitate E2 from VEEV-infected cells. Our results show that antibodies against E2 immunoprecipitated actin (more than 8-fold increase by densitometry analysis) and antibodies against actin immunoprecipitated E2 (more than 4-fold increase by densitometry analysis) from VEEV-infected, but not from mock-infected cells (Fig 6D right panel). These results indicate that VEEV E2 either directly or indirectly associates with actin in lysates from infected cells. However, since our lysis buffer included detergent (NP-40), the observed association between E2 and actin was most likely not in the context of CPV-II structures.

### Actin, Rac1, and Arp3 Inhibitors Interfere with Alphavirus E2 Trafficking from TGN to the Cell Surface

**Fig 7:**
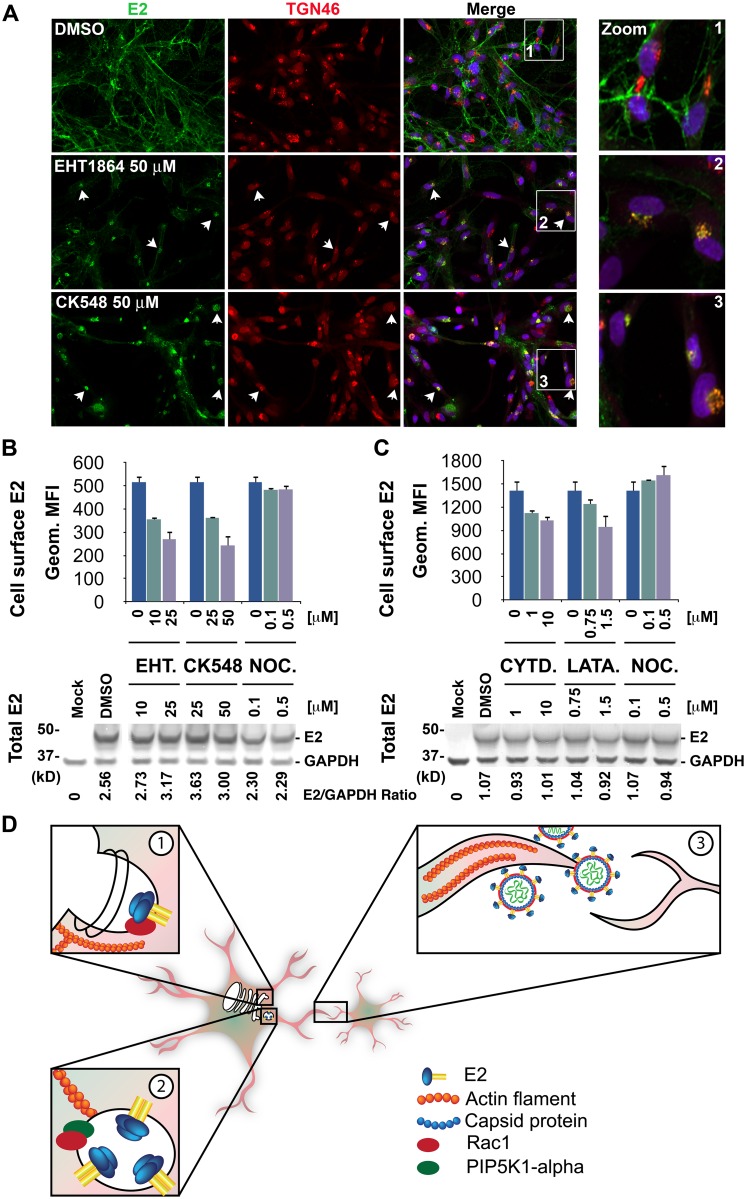
Actin, Rac1, and Arp3 inhibitors block E2 transport to cell surface. (**A**) Representative confocal images of primary human astrocytes treated with DMSO, EHT1864, or CK548 and subsequently infected with VEEV (MOI = 0.005) for 18 h. Cells were stained with VEEV E2 (green)- and TGN46-specific antibodies (red), and a nuclear stain (blue). Representative cells showing co-localization of E2 and TGN46 are indicated with white arrows. (**B** and **C**) Upper panels: Geometrical mean fluorescent intensity of cell-surface E2 staining in HeLa cells infected with VEEV TC-83 (MOI = 10) and treated with EHT1864, CK548, cytochalasin D, latrunculin A, or nocodazole as measured by flow cytometry. HeLa cells were infected with VEEV TC-83 for 5 h and subsequently treated with increasing concentrations of the inhibitors or DMSO (control). Five (**B**) or six (**C**) h later cells were dissociated and stained against VEEV E2 and with a 7-amino-actinomycin D viability dye. Bottom panel: Immunoblot of total E2 expression in whole cell lysates of HeLa cells described in (**B**) and (**C**). GAPDH was used as a loading control. Densitometric analysis of western blots was performed with ImageJ. (**D**) Model for trafficking of alphavirus E2 from the TGN to the cell surface. (1) Biogenesis of vacuoles (CPV-II) containing E1/E2 at the TGN is dependent on ADP-ribosylation factor 1 (Arf1) and Rac1. (2) CPV-II containing E1/E2 traffic to the cell surface via actin by a Rac1- and Arp3-dependent mechanism. Rac1 and PIP5K1-α are also localized to these actin filaments. (3) Actin tunneling nanotubes mediate alphavirion spread to neighboring cells.

E2 was mainly localized in perinuclear puncta in cells treated with the Rac1 and Arp3 inhibitors, whereas in DMSO-treated cells E2 was found throughout the cytoplasm and at the plasma membrane (Fig 5D). Previous studies have demonstrated that the alphavirus glycoproteins E1/E2 are transported from the TGN to the cell surface via TGN-derived vacuoles [12,35], suggesting that the observed puncta might represent TGN or TGN-derived vacuoles. We therefore hypothesized that Rac1- and Arp3-dependent actin remodeling in alphavirus-infected cells might be important for trafficking of E1/E2. To test this hypothesis, primary human astrocytes were treated with DMSO, EHT1864, or CK548 and then infected with VEEV. Cells were stained with antibodies against VEEV E2 glycoprotein and the TGN marker TGN46. VEEV E2 was primarily located at the cell surface in control DMSO-treated cells (Fig 7A, zoom 1). In some of the cells, E2 puncta co-localized with TGN46. However, upon treatment with the Rac1 or Arp3 inhibitors, E2 localization in TGN46-positive puncta was significantly enhanced (Fig 7A, zoom 2 and 3) and less E2 glycoprotein was observed at the cell surface. Quantification of TGN46-to-cell-surface ratio of E2 staining intensity in control- or compound-treated VEEV-infected astrocytes is shown in S7A Fig. Similar experiments performed in HeLa cells using the inhibitors and the TGN marker Golgi-localized, gamma ear-containing, ARF-binding protein 3 (GGA3) yielded comparable results (S7B Fig).

In addition, we developed a flow cytometry-based assay for detection of VEEV E2 on the plasma membrane. We examined cell-surface expression of E2 following treatment with actin polymerization, microtubules depolymerization, Rac1, or Arp3 inhibitors. HeLa cells were infected with VEEV and treated 5 h later with increasing concentrations of EHT1864, CK548, latrunculin A, cytochalasin D, or nocodazole. Cells were subsequently stained for surface expression of E2 and with the 7-amino-actinomycin D viability dye. Concomitantly, an aliquot of the cells of each treatment group was lysed and analyzed for total E2 expression in whole-cell lysates. None of the inhibitors significantly affected total protein levels of E2. However, the actin, Rac1, and Arp3 inhibitors decreased geometric mean fluorescence intensity of E2 on the cell surface in a dose-dependent manner (Fig 7B and 7C). In contrast, the microtubule inhibitor, nocodazole, had no effect on cell surface E2 expression. The effect of the actin, Rac1, and Arp3 inhibitors on E2 surface expression was specific as no or minimal effect was observed on surface expression of cellular CD44 (S7C Fig). Overall, our data suggest that actin, Rac1, and Arp3, but not microtubule, inhibitors might interfere with trafficking of E2 from the TGN or TGN-derived vacuoles to the cell surface.

### Localization of VEEV-Induced Actin Foci, Rac1, and PIP5K1-α in Relation to the TGN

To examine if the actin remodeling observed in alphavirus-infected cells is associated with any TGN membrane structures, we stained VEEV-infected cells with the TGN marker TGN46. Actin clusters were observed near TGN46 (S8A Fig) and VEEV E2 was detected on these actin clusters and co-localized with the TGN marker. Rac1 was also found to co-localize with the TGN marker and E2, whereas PIP5K1-α co-localized with E2 but not with TGN46 (S8B and S8C Fig).

## Discussion

Reorganization of the host cytoskeleton varies among infections with different viruses and can play a role in every stage of the viral life cycle. Examples include virion movement (surfing) towards entry sites, actin-enhanced endocytic entry pathways, and actin-based, filopodial extensions (termed tunnelling nanotubes) that act as bridges to facilitate virus spread (reviewed in [36–39]).

Here, using an siRNA screen, we identified trafficking host factors that are important for alphavirus infection and are crucial regulators of the actin cytoskeleton. To date, Rac1- and Arp2/3-mediated actin rearrangements have mainly been associated with virus uptake and entry [30,40–44]. Rac1 is predominantly known as a key regulator of the actin cytoskeleton at the plasma membrane [45]. There, Abelson interactor 1 (Abi1) and Wiskott-Aldrich syndrome protein (WASP) family verprolin-homologous protein (WAVE), but not neural (N)-WASP, are essential for Rac1-dependent membrane protrusion and macropinocytosis [46].

Recently, however, Rac1, the Arp2/3 complex, and actin have emerged as major factors in the secretory pathway in processes such as biogenesis and motion of Golgi-derived transport carriers to the plasma membrane [47–50]. During formation of TGN carriers, Rac1 functions downstream of ADP-ribosylation factor 1 (Arf1). Arf1 recruits clathrin/adaptor protein 1 (AP-1)-coated carriers and a complex composed of cytoplasmic fragile X mental retardation 1 (FMR1)-interacting protein (CYFIP), nucleosome assembly protein 1 (NAP1), and Abi1 to the TGN. Rac1 and its exchange factor Rho guanine nucleotide exchange factor 7 (ARHGEF7) bind CYFIP and trigger N-WASP- and Arp2/3-mediated actin polymerization necessary to tubulate clathrin-AP-1-coated carriers [51]. Therefore, during alphavirus infection, Rac1 could potentially be recruited to the TGN to mediate biogenesis of E2-containing vesicles and/or their transport from the TGN to the cell surface via actin (see model, Fig 7D). In support of this hypothesis, some of the host factors mentioned above, such as clathrin heavy chain 1 (CLTC), AP-1 subunits (AP1M1), and Arf1 were identified as hits in our primary and validation siRNA knockdown screens (S1 and S2 Tables). Furthermore, siRNAs targeting N-WASP reduced the infection rate of both VEEV and CHIKV (S1B Fig). Finally, during VEEV infection Rac1 was found to co-localize with E2 at the TGN (S8 Fig). Hence, Arf1 may function upstream of Rac1 to facilitate biogenesis and/or motion of E2 transport carriers from the TGN to the plasma membrane and that this transport is mediated by N-WASP.

Viruses have evolved specific egress pathways for transporting viral components to the plasma membrane, often using the cell’s secretory pathway via the endoplasmic reticulum, the Golgi, and even transport vesicles. Most exocytic transport of cellular secretory cargo to the plasma membrane relies on microtubules for long-range translocations [52,53]. The microtubule network is also emerging as the preferred cytoskeletal element recruited for transportation of components of certain viruses to the cell surface [54–57]. Examples are microtubule delivery of influenza A virus HA membrane glycoprotein to the apical surface of MDCK cells [58] and vesicular stomatitis Indiana virus glycoprotein G trafficking from the TGN-to-plasma membrane [59].

In contrast, our results demonstrate that transport of the alphavirus membrane glycoprotein E2 is at least in part dependent on actin and actin regulators (Rac1 and Arp3). We hypothesize that the coordinated activities of PIP5K1-α, Rac1, and the Arp2/3 complex might mediate alphavirus envelope E2 trafficking from the TGN to the cell surface via actin. Several results support this actin-dependent transport model (Fig 7D). First, time-of-addition experiments with Rac1 and Arp3 inhibitors demonstrated that both factors function at a late stage of virus infection (Fig 3). Second, within a similar time frame (concomitantly with E2 expression in infected cells) major actin rearrangements into clusters occur in alphavirus-infected cells (Figs 3 and 5). Super high-resolution fluorescence microscopy and electron microscopy show that CPV-II structures containing E1 or E1/E2, respectively, are localized along or at the end of actin filaments. Rac1 and PIP5K1-α also co-localize with E2 on actin foci (Fig 5). In infected cell lysates, E2 envelope protein was found to associate (either directly or indirectly) with actin (Fig 6). Third, Rac1 and Arp3 inhibitors blocked formation of virus-induced actin clusters (Fig 5). In cells treated with actin, Rac1, or Arp3 inhibitors, most of the E2 staining was found to localize with TGN markers, and E2 levels at the cell surface were reduced (Fig 7). We have not yet examined the role of actin, PIP5K1-α, Rac1, and the Arp2/3 complex in E1 trafficking. However, since E1 and E2 are oligomerized into trimeric complexes during transit to the plasma membrane in CPV-II structures [60], we speculate that these host factors will have a similar function in trafficking of both viral proteins.

Actin dynamics are involved in numerous aspects of intracellular transport. However, little is known regarding manipulation of these host machineries by pathogenic alphaviruses. Viruses can serve as unique tools to decipher how a particular cargo recruits actin filament tracks and the host factors and motors associated with these movements. Our results suggest a previously unidentified role of host factors Rac1, Arp3 and PIP5K1-α late in alphavirus infection via actin remodeling that possibly mediates transport of alphavirus envelope glycoproteins from the TGN to the cell surface. It is important to note that although our data indicate that actin plays a major role in alphavirus glycoprotein transport, our experiments do not exclude the existence of other, parallel, transport mechanisms mediated by intermediate filaments or microtubules.

Recombinant alphaviruses expressing tagged E2 could be useful to further substantiate our findings. However, until now, we have not succeeded in rescuing such viruses. Finally, our high-content siRNA screen reveals novel host regulators of alphavirus infection and potential therapy targets.

## Materials and Methods

### siRNA Screens

An arrayed library targeting 140 trafficking genes (Dharmacon Human ON-TARGETplus siRNA Library—Membrane Trafficking—SMARTpool, G-105500-05, Thermo Scientific,) was used to transiently reverse-transfected HeLa cells (10,000 cells per well, 96-well format) in triplicate at a 30-nM final concentration, using HiPerfect (Qiagen). Cells were washed on the following day and infected 24 h later with VEEV ICS-SH3 at an MOI of 0.5 for 20 h. Cells were fixed with 10% formalin (Val Tech Diagnostics) and stained for high-content quantitative image-based analysis. The screen was repeated three times. In 6 wells on each plate, cells were transfected with a negative control siRNA (NT, siCONTROL Non-Targeting siRNA #2, Dharmacon D-001210-02). The infection rate of control siRNA-transfected cells was optimized to yield, on average, 70–80%, following multiple virus replication cycles.

For the primary screen, siRNA pools were classified as hits if the average of triplicate wells showed that the percentage of VEEV-positive cells decreased by more than 30% compared to that observed with the control siRNA wells on the plate (Z-score <-2 SD). In the validation screen, the individual oligomers comprising each pool were placed into separate wells, and the screen was repeated. siRNA targets were considered validated if two or more of the individual oligomers were classified as hits compared to the control wells on the plate (similar parameters as above) and if the cell number was not less than 30% of the average of the negative control wells on the plate. Catalog numbers and sequences of siRNAs are provided in Table 1. The percent of infected cells relative to controls, as well as the normalized cell numbers (normalized to control siRNA) is provided in S1 Table.

**Table 1:** Sources of Human-sequence Reagents.

cDNA/Gene	Primer Function	Catalog Number/ Sequence	Vendor
RAC1-1	siRNA	J-003560-14/ GUGAUUUCAUAGCGAGUUU	Dharmacon/Thermo Scientific
RAC1-2	siRNA	J-003560-15/ GUAGUUCUCAGAUGCGUAA	Dharmacon/Thermo Scientific
RAC1-3	siRNA	J-003560-16/ AUGAAAGUGUCACGGGUAA	Dharmacon/Thermo Scientific
RAC1-4	siRNA	J-003560-17/ GAACUGCUAUUUCCUCUAA	Dharmacon/Thermo Scientific
RAC1-5	siRNA	s11711	Applied Biosystems/Life Technologies
RAC1-6	siRNA	s117112	Applied Biosystems/Life Technologies
RAC1-7	siRNA	s11713	Applied Biosystems/Life Technologies
ACTR3-1	siRNA	J-012077-06/ GCAGUAAAGGAGCGCUAUA	Dharmacon/Thermo Scientific
ACTR3-2	siRNA	J-012077-07/ GUGAUUGGCAGCUGUAUUA	Dharmacon/Thermo Scientific
ACTR3-3	siRNA	J-012077-08/ GGAAUUGAGUGGUGGUAGA	Dharmacon/Thermo Scientific
ACTR3-4	siRNA	J-012077-09/ GCCAAAACCUAUUGAUGUA	Dharmacon/Thermo Scientific
ACTR3-5	siRNA	s19640	Applied Biosystems/Life Technologies
ACTR3-6	siRNA	s19641	Applied Biosystems/Life Technologies
ACTR3-7	siRNA	s19642	Applied Biosystems/Life Technologies
PIP5K1A-1	siRNA	J-004780-09/ ACACAGUACUCAGUUGAUA	Dharmacon/Thermo Scientific
PIP5K1A-2	siRNA	J-004780-10/ GCACAACGAGAGCCCUUAA	Dharmacon/Thermo Scientific
PIP5K1A-3	siRNA	J-004780-11/ GUGGUUCCCUAUUCUAUGU	Dharmacon/Thermo Scientific
PIP5K1A-4	siRNA	J-004780-12/ GUAAGACCCUGCAGCGUGA	Dharmacon/Thermo Scientific
PIP5K1A-5	siRNA	s15932	Applied Biosystems/Life Technologies
WASL-1	siRNA	s17132	Applied Biosystems/Life Technologies
WASL-2	siRNA	s17133	Applied Biosystems/Life Technologies
WASL-3	siRNA	s17134	Applied Biosystems/Life Technologies
PPIB	qRT-PCR	Hs00168719_m1	Applied Biosystems/Life Technologies
PIP5K1A	qRT-PCR	Hs00801004_s1	Applied Biosystems/Life Technologies
TC83 forward primer	qRT-PCR	CTTGGCAAACCTCTGGCAGC	Life Technologies
TC83 Probe	qRT-PCR	6FAM-CTCTTCATgCAATgCCCTTCTCCTgTCA	Life Technologies
TC83 reverse primer	qRT-PCR	ATACCCACTCggTTCCAgCg	Life Technologies
Rac1 K186E forward primer	Site-directed mutagenesis	5'-CCC GCC TCC CGT GAA GAA GAA GGA GAG AAA ATG CC-3'	Integrated DNA Technologies
Rac1 K186E reverse primer	Site-directed mutagenesis	5'-GGC ATT TTC TCT CCT TCT TCT TCA CGG GAG GCG GG-3'	Integrated DNA Technologies

### Cell Lines and Plasmid Constructs

HeLa (ATCC, #CCL-2), BHK-21 (ATCC, #CCL-10), and Vero cells (ATCC, #CCL-81) were maintained in Eagle’s minimum essential medium supplemented with 10% fetal calf serum. T-REx-HeLa cells expressing human wild type Rac1 fused to eGFP, and Flp-In 293 T-REx cells expressing human wild type Rac1, Rac1 G12V, Rac1 T17N, Rac1 K186E or CAT upon tetracycline induction were generated by using the T-REx System or the Flp-In T-REx system, respectively, according to the manufacturer's instructions (Life Technologies). Cells were induced to express wild-type human Rac1, variants thereof, or CAT in 96-well plates by adding tetracycline (1 μg/ml) to the growth medium. Normal human astrocytes were obtained from Lonza and maintained according to the provider's instructions. Plasmids encoding Rac1 variants (wild-type Rac1, Rac1 T17N or Rac1 G12V) fused to an avian myelocytomatosis (myc) protein tag were purchased from the Missouri S&T cDNA Resource Center (www.cdna.org). A plasmid encoding Rac1 K186E was generated by using the QuikChange Lightning Site-Directed Mutagenesis Kit (Agilent Technologies). Sequences of the primers are provided in Table 1. A plasmid encoding pcDNA3-EGFP-Rac1-wt was obtained from Addgene.

### Antibodies, Dyes, and Pharmacological Inhibitors

Mouse monoclonal antibodies against CHIKV (2D21-1), EEEV (1C2), VEEV (1A4A-1), WEEV (9F12), and RVFV envelope glycoprotein Gn (4D4) and nucleoprotein (R3-ID8-1-1) were obtained from US Army research Institute of Infectious Diseases (USAMRIID) archives [61]. Goat antibody against VEEV capsid (C) or envelope protein was generously provided by AlphaVax (via Kurt Kamrud). Rabbit antibodies against Arp3, actin, N-WASP, GAPDH, FLAG, and HA were obtained from Sigma-Aldrich. Mouse monoclonal antibodies against actin, CD44, GGA3, and Rac1 were purchased from BD Transduction Laboratories. Rabbit antibody against α/β-tubulin was obtained from Cell Signaling Technology. Sheep anti-human TGN46 antibody was from AbD Serotec. Alexa Fluor-conjugated antibodies and phalloidin, Hoechst 33342, and HCS CellMask Red were obtained from Life Technologies. All chemical inhibitors were purchased from Sigma-Aldrich, with the exception of EHT1864 (Tocris Bioscience). Cells were incubated with inhibitors for 1 h before addition of viruses unless otherwise indicated in the figure legends.

### Virus Infections, Viral Transduction, and Replicon Assays

Infections with VEEV IC-SH3, EEEV FL91-4679, WEEV CBA87, RVFV ZH501, and CHIKV AF15561 were conducted under Biosafety Laboratory 3 conditions. All alphaviruses were propagated in BHK-21 cells and purified via sucrose gradients. RVFV was propagated in Vero cells. Viral infectivity was titrated by plaque assays as previously described [62].

MoMLV-eGFP pseudotypes carrying the VEEV envelope proteins E1/E2 or Ebola virus envelope GP_1,2_ (control) were produced as previously described [27,28,63]. MoMLV-eGFP pseudotypes were added to siRNA-treated HeLa cells for 6 h. Cells were then washed and supplemented with growth medium. Cell transduction efficiency was determined 2 days later by measuring eGFP expression using an Opera confocal reader (PerkinElmer).

For CHIKV replicon assays, we used the previously described BHK-CHIKV-NCT cells, which contain the CHIKV replicon with two reporter genes, Renilla luciferase (*Rluc*) and *EGFP* [31].

BHK-CHIKV-NCT cells were seeded onto 96-well plates at densities of 2 × 10^4^ cells/well, incubated overnight, and treated with the indicated compounds at various concentrations. After exposure for 48 h, the Rluc activity resulting from the translation of CHIKV-*Rluc* genomic RNA was determined from the lysates using a Rluc assay kit (Promega) with a Tecan microplate reader.

### Immunoprecipitation and Western Blot Analyses

HeLa cells in 6-well plates were infected with VEEV TC-83 or RVFV MP12 (MOI = 1) for 8 h. Cells were lysed in a mild lysis buffer (50 mM Tris pH 7.4, 50 mM NaCl, 0.2 mM ethylenediaminetetraacetic acid (EDTA), and 1% Triton X-100) or a lysis buffer (25 mM Tris pH 7.4, 150 mM NaCl, 1 mM EDTA, 5% glycerol, and 1% NP-40) from Pierce Crosslink Immunoprecipitation supplemented with Complete protease inhibitor cocktail (Thermo Scientific Pierce). Cleared lysates were incubated overnight at 4°C with protein A/G beads (Thermo Scientific Pierce) and VEEV E2- or RVFV Gn-specific antibodies or with beads cross-linked to antibodies against VEEV E2 or actin. Cell lysate immunoprecipitates were analyzed by SDS-PAGE and immunoblotting using the indicated antibodies.

For western blot analyses, cells were lysed with RIPA lysis and extraction buffer supplemented with complete protease inhibitor cocktail (Thermo Scientific Pierce). Cleared lysates were analyzed by SDS-PAGE and immunoblotting using WesternBreeze chromogenic or chemiluminescent kits (Life Technologies) and the indicated antibodies. Densitometric analysis of western blots was performed with ImageJ [64].

### Stimulated Emission Depletion Microscopy (STED)

Cells were grown on glass cover slips and inoculated with VEEV or CHIKV for 1 h. Cells were fixed 20 h (VEEV) or 48 h (CHIKV) later, permeabilized with 0.5% Triton X-100 (Sigma-Aldrich) in phosphate-buffered saline (PBS), blocked with 3% bovine serum albumin in PBS for 1 h. and stained using mouse anti-E2 antibodies (1:1,000 dilution), followed by ATTO 647N Goat Anti-Mouse IgG (Active Motif) (1:2,000 dilution). Actin was stained with Phalloidin ATTO 565 (Sigma-Aldrich) (1:80 dilution). Slides were mounted in ProLong Gold Antifade Reagent (Life Technologies) and dried overnight at room temperature before imaging. All confocal images were acquired on the Leica SP5 TCS 2C STED confocal system (Leica Microsystems) equipped with Leica’s inverted DMI 6000 microscope and STED 100x oil objective. Images were acquired at an imaging speed of 400 Hz, pin hole set to Airy1, line average of 6, and 1024 X 1024 formats. For STED of ATTO dyes, the pulsed Ti:SA infra red laser (Mai Tai, model # MAI TAI BB990, Spectra-Physics) was tuned to 740 nm.

### Electron Microscopy

HeLa cells grown on a MatTek dish (MatTek corporation, MA) were infected with VEEV TC83 (MOI = 5) for 20 h. Cells were fixed for 1 h in primary fixative (2.5% formaldehyde, 2.5% glutaraldehyde, 0.1 M sodium cacodylate, pH 7.4), washed three times in ice-cold 0.1 M sodium cacodylate buffer, and incubated with 1% osmium tetroxide in 0.1 M of sodium cacodylate for 1 h, washed three times with distilled water, stained and stabilized on ice with 2% uranyl acetate for 1 h and successively dehydrated on ice through a series of 22%, 50%, 75%, and 95% ethanols. The cells were then dehydrated three times at room temperature in 100% ethanol and infiltrated in well-mixed 50% ethanol and 50% Durcupan ACM resin (Fluka, Sigma-Aldrich) for 1 h with agitation. Cells were infiltrated twice by 100% Durcupan ACM for 3 h with agitation, after which the samples were placed in an oven and polymerized at 60°C for at least 48 h. The glass coverslip was peeled away from the bottom using a razor blade, and the selected area was cut out and glued to a block for sectioning. Thin sections (approximately 80 nm) were collected and pre-stained with 1% uranyl acetate and Sato lead before examination on a JEOL 1011 transmission electron microscope at 80 kV. Digital images were acquired using an AMT camera system.

### Immunofluorescence and High-Content Quantitative Image-Based Analysis

Plasmid encoding HA-tagged PIP5K1α was generously provided by Dr. Richard Anderson (University of Wisconsin). Plasmid encoding FLAG-tagged nsP1 was generated in-house by PCR. Plasmids were transiently reverse-transfected into HeLa cells on glass coverslips (Fisher Scientific) using Lipofectamine LTX Reagent (Life Technologies). T-REx HeLa cells on glass coverslips were induced with tetracycline for 24 h to express Rac1-eGFP. VEEV-infected cells were fixed, permeabilized, and blocked as described for STED. After incubation with primary antibodies and fluorescent secondary antibodies, slides were mounted as described for STED and air-dried before imaging with a TCS-SP5 confocal/multiphoton microscope (Leica Microsystems). All confocal images represent a single plane acquired with a 100× oil objective. Similar experimental conditions were used for imaging studies of actin, tubulin, TGN46, and VEEV E2 in HeLa cells. Co-localization analysis of tubulin or actin with VEEV E2 was performed with the ImageJ program using the Interactive 3D Surface Plot plugin [64].

For analysis of the siRNA screen, cells were stained without prior permeabilization. Cells inoculated with CHIKV, EEEV, RVFV, WEEV or SINV or cells designated for phalloidin or TGN staining were permeabilized prior to blocking as described above. Cells were then stained with murine monoclonal antibodies against the indicated viral proteins (1:1,000 dilution) and, where indicated, against TGN46 or GGA3 (1:250 dilution). Subsequently, cells were stained with appropriate Alexa Fluor-conjugated antibodies (1:1,000 dilution), and Alexa Fluor 568 Phalloidin (1:100 dilution) where indicated. All infected cells were also stained with Hoechst 33342 and HCS CellMask DeepRed for nuclei and cytoplasm detection, respectively.

High-content quantitative imaging data were acquired and analyzed on an Opera quadruple excitation high sensitivity confocal reader (model 3842 and 5025; Perkin-Elmer), at two exposures using a ×10 air, ×20 water, or ×40 water objective lenses as described in [65]. Images were analyzed using Acapella 2.0, 2.6, 2.7 (Perkin-Elmer) scripts in Evoshell or the building-blocks interface in the Columbus image analysis server (PerkinElmer). Nuclei and cytoplasm staining were used to determine total cell number and cell borders, respectively. Mock-infected cells were used to establish a fluorescence intensity threshold for virus-specific staining. Quantification of virus-positive cells was subsequently performed based on mean fluorescent intensities in the virus-specific staining channel. Infection rates were then determined by dividing the number of virus-positive cells by the total number of cells measured. Detailed pipelines for image-based quantification of alphavirus-induced actin foci and TGN46-to-plasma membrane E2 staining intensity ratio are available upon request. At least 5,000 cells and up to 15,000 cells were analyzed per replicate in drug- or siRNA-treated cells. For actin foci analysis, 1,000–1,500 cells were used per replicate. For analysis of TGN46-to-plasma membrane E2 staining intensity ratio, 700 cells were used per replicate.

### Flow Cytometry

HeLa cells in 12-well plates were inoculated with VEEV TC-83 (MOI = 10) for 5 h. DMSO, EHT1864, CK548, nocodazole, latrunculin A, or cytochalasin D were subsequently added at the indicated concentrations. Five or 6 h later, cells were detached with Cell Dissociation Buffer (Life Technologies) and washed with flow buffer (PBS/0.5% bovine serum albumin/2mM EDTA). Cells were incubated with mouse anti-VEEV E2 or CD44 primary antibody (1:1,000 dilution in flow buffer) for 30 min on ice and then washed twice with ice-cold flow buffer. Cells were incubated for 20 min in the dark with Alexa Fluor 488 Goat Anti-Mouse IgG secondary antibody (Life Technologies) (1:5,000 dilution in ice-cold flow buffer) and with 7-amino-actinomycin D to exclude dead cells from analysis (1:500 dilution). Following two more washes with ice-cold flow buffer, cells were fixed in 1% paraformaldehyde. Cytometric collection was performed using a FACS Canto II (BD Biosciences), and data were analyzed using Flowjo v7.6.5 (TreeStar).

### qRT-PCR

VEEV TC-83 RNA yields from the media and the cells and relative expression levels of PIP5K1-α in siRNA-treated HeLa cells were determined by qRT-PCR as previously described [65]. Serial 10-fold dilutions of the assayed (10^2^ to 10^7^ copies) virus were used as standards. Relative expression levels were determined by using the comparative cycle threshold method. Sequences of qRT-PCR probes/primers are provided in Table 1.

### Statistical Analysis

Data are representative of at least three independent experiments, and values are given as mean of triplicates ± standard deviation (SD) unless otherwise indicated. Statistical significance was determined by the paired Student’s *t* test.

## Supporting Information

S1 Table Summary of the primary siRNA screen. The primary screen was performed using a pool of four siRNA duplexes per gene from a Dharmacon Membrane Trafficking library. The measured effects of each annotated siRNA pool on VEEV infection rate and cell number were normalized to that observed with control siRNA.
(XLSX)

S2 Table Summary of the follow-up siRNA screen. For the deconvolution screen, the four individual duplexes siRNAs from each siRNA pool were used. The measured effects of each annotated siRNA duplex on VEEV infection rate and cell number were normalized to that observed with control siRNA.
(XLSX)

S1 Fig siRNA screen identifies host regulators of alphavirus infection. (**A**) High-content quantitative image-based analysis was used to measure relative infection rates (normalized to control siRNA-treated cells) of CHIKV in HeLa cells pretreated with the indicated siRNAs. Cells were infected for 24 h (CHIKV, MOI = 5), fixed and stained with antibodies against E2. (**B**) HeLa cells were pretreated with the indicated siRNAs and infected for 20 h with VEEV (MOI = 0.5) or for 24 h with CHIKV (MOI = 5). Cells were fixed, stained, and analyzed as in (**A**). Protein levels of N-WASP and actin (loading control) following siRNA treatment were determined by immunoblotting (right panel). Values represent the mean ± SD, n = 3.
(TIF)

S2 Fig Rac1, Arp3, and formation of a Rac1:PIP5K1-α complex are important for alphavirus infection. (**A**) Primary human astrocytes were treated with increasing concentrations of CK548 and subsequently infected with EEEV or WEEV (MOI = 0.005). Cells were fixed in formalin 19 h after infection, stained with virus-specific antibodies, and analyzed using an Opera confocal imager. Results are normalized to DMSO-treated samples. (**B**) HeLa cells were treated with CK548 or EHT1864 and subsequently infected with CHIKV or SINV (MOI = 5). Cells were fixed 20 h (SINV) or 48 h (CHIKV) later and analyzed as in (**A**). (**C**) Representative confocal images of (Fig 2F). VEEV E2 glycoprotein staining is shown in green and nucleus/cytoplasm staining is shown in red. (**D**) Flp-In T-REx 293 cells pre-induced to express chloramphenicol acetyltransferase (CAT), wild-type Rac1, or variants thereof were infected with VEEV (MOI = 0.1). After 18 h, virus titer in the supernatants was determined by plaque assay. **, *p* < 0.01, Student's *t* test (between samples and CAT). (**E**) Representative confocal images of (Fig 2H). Coloring as in (**C**). (**F**) Confocal images of Flp-In T-REx 293 cells that were induced as in (**D**), inoculated with WEEV (MOI = 0.005), fixed 18 h later, and stained with virus-specific antibodies (green) and nuclear stain (blue). (**G**, **H**) High-content quantitative image-based analysis of CHIKV infection rates in Flp-In T-REx 293 cells pre-induced as in (**D**). Cells were fixed 24 h after virus inoculation and stained with virus-specific antibodies. (**I**) Representative confocal images of (**G**, **H**). CHIKV E2 glycoprotein staining is shown in green and nucleus/cytoplasm staining is shown in red. All values represent the mean ± SD, n = 3.
(TIF)

S3 Fig Rac1 and Arp3 act at a late stage of alphavirus infection. (**A**) Time course of VEEV TC-83 (MOI = 10) infection in HeLa cells. Media containing extracellular virus were harvested at the indicated time points for qRT-PCR analysis of virion copy number (left panel). Infected cells were fixed, stained with VEEV E2-specific antibody, and analyzed with an Opera confocal reader by high-content quantitative image-based analysis (right panel). (**B**) High-content quantitative image-based analysis of relative VEEV TC-83 infection rates (normalized to DMSO-treated samples) in time-of-addition experiments. VEEV-infected HeLa cells (MOI = 1) were treated with increasing concentrations of the Rac1 inhibitor EHT1864, or the Arp3 inhibitor CK548 at the indicated time points prior to (-1 h) or after (+1–7 h) virus addition. Cells were fixed 12 h after addition of virus and stained with virus-specific antibodies. Values represent the mean ± SD, n = 3. (**C**) Plaque assays were used to measure VEEV titer in supernatants of infected HeLa cells treated with the indicated concentrations of the inhibitors. Cells were treated with inhibitors 5 h after inoculation with VEEV (MOI = 0.5), and virus-containing media was harvested for analysis 17 h later. Values represent the mean ± SD, n = 3. **, *p* < 0.01, Student's *t* test (between samples and DMSO). (**D**) BHK-CHIKV-NCT cells expressing a CHIKV replicon with a *Renilla* luciferase reporter were treated with increasing concentrations of EHT1864, CK548, or T705 (a nucleotide prodrug, positive control). After 48 h, *Renilla* luciferase (Rluc) activity was determined from the lysates.
(TIF)

S4 Fig Actin polymerization plays a role at a late stage of alphavirus infection. (**A**) HeLa cells or primary human astrocytes were infected with VEEV (MOI = 0.5) or VEEV TC-83 (MOI = 0.005) for 3 h (HeLa) or 5 h (astrocytes) and then treated with increasing concentrations of nocodazole. After 6 h (astrocytes) or 17 h (HeLa), virus titer in the supernatants was determined by plaque assay. Values represent the mean ± SD, n = 3. (**B-C**) Representative confocal images of (Fig 4C). VEEV E2 staining is shown in green, nucleus staining is shown in blue, and tubulin (**B**) or actin (**C**) staining is shown in red (top panel: magnification: 10x; bottom panel: magnification: 40x). (**D**) BHK-CHIKV-NCT cells expressing a CHIKV replicon with a *Renilla* luciferase reporter were treated with increasing concentrations of the indicated inhibitors. After 48 h, *Renilla* luciferase (Rluc) activity was determined from the lysates.
(TIF)

S5 Fig Alphavirus infection causes actin rearrangements into actin foci that co-localize with Rac1, PIP5K1-α, and E2. (**A**) HeLa cells were inoculated with WEEV (MOI = 2), EEEV (MOI = 1), or SINV (MOI = 5), fixed 24 h later, and stained with virus-specific antibodies and fluorescent phalloidin. High-content quantitative image-based analysis was used to measure virus infection rates (left panel) as well as number of actin foci per cell (right panel). ***, *p* < 0.0001, Student's *t* test (between samples and mock). (**B**) HeLa cells were transfected with expression plasmids encoding VEEV nsP1-FLAG. Cells were fixed 24 h later and stained with antibodies against FLAG (green), and fluorescent phalloidin (red). Confocal images of single Z sections and a Z stack image (merged Z sections) are shown of actin staining. Zoom on actin filopodia indicated by a white arrow is shown (right panel). Representative actin filopodia are indicated by asterisks. (**C-D**) Basal-to-apical confocal section series of VEEV-infected HeLa cells (MOI = 5). Co-localization of HA-tagged PIP5K1-α (**C**) or Rac1 (**D**) (blue), actin (red), and VEEV E2 (green), at different Z sections is shown. Insets: zoom on actin filaments indicated by white arrows. Nuclei are indicated by asterisks. Single channel intensities were measured along lines crossing different actin clusters (right panels). VEEV was added to (**C**) HeLa cells that were reverse-transfected with a plasmid encoding HA-tagged PIP5K1-α or (**D**) tetracycline-induced T-Rex HeLa cells that expressed Rac1 fused to eGFP. Cells were fixed 20 h later, permeabilized, and stained with VEEV E2-specific antibody, phalloidin, and an antibody against HA (**C**).
(TIF)

S6 Fig Alphavirus E2 co-localizes with actin but not with tubulin. (**A**) Representative images of VEEV-infected HeLa cells from Fig 6A in confocal and STED microscopy modes. E2 glycoprotein is shown in green and actin in red. (**B**) Co-localization of tubulin (blue), actin (red), and E2 (green) in a VEEV-infected cell at different Z sections from base (Section 7) to apex (section 25). HeLa cells were infected with VEEV (MOI = 5) for 20 h and stained with antibodies against E2, tubulin, and fluorescent phalloidin. Pixel intensities of tubulin (red) and E2 (green) staining are shown (bottom graphs). (**C**) Representative images of VEEV-infected HeLa cells (as in **B**) stained with E2-specific antibodies (green), phalloidin (red) and CellMask (grey in merge). Analysis of cell borders based on CellMask staining was performed within the Columbus programming environment.
(TIF)

S7 Fig Rac1 and Arp3 inhibitors block E2 transport to cell surface. (**A**) High-content quantitative image-based analysis was used to measure the TGN46-to-plasma membrane E2 staining intensity ratio in VEEV-infected astrocytes. *, *p* < 0.05, **, *p*< 0.001, Student's *t* test (between samples and DMSO). (**B**) Representative confocal images of HeLa cells treated with DMSO, EHT1864, or CK548 at the indicated concentrations and subsequently infected with VEEV (MOI = 0.5). Cells were fixed and stained with VEEV E2 (green) and GGA3 (red)-specific antibodies and counterstained with Hoechst 3342 (blue) 20 h after infection (magnification: 40x). Representative cells showing co-localization of E2 and GGA3 are indicated with white arrows. (**C**) Geometrical mean fluorescent intensity of cell-surface CD44 staining in HeLa cells treated with EHT1864, CK548, cytochalasin D, latrunculin A, or nocodazole as measured by flow cytometry. HeLa cells were treated with increasing concentrations of the inhibitors or DMSO (control). Six h later cells were dissociated and stained against CD44 and with a 7-AAD viability dye.
(TIF)

S8 Fig Localization of actin foci, Rac1, and PIP5K1-α relative to TGN46. VEEV (MOI = 5) was added to (**A**) HeLa cells, or (**B**) tetracycline-induced T-Rex HeLa cells that express Rac1 fused to eGFP or (**C**) HeLa cells that were reverse-transfected with a plasmid encoding HA-tagged PIP5K1-α. Cells were fixed 20 h later, permeabilized, and stained with VEEV E2- and TGN46-specific antibodies, as well as with phalloidin (**A**), and an antibody against HA (**C**). Co-localization of actin (**A**), Rac1 (**B**), or PIP5K1-α (**C**) with TGN46 (white), and VEEV E2 (green), at a single Z section.
(TIF)
