# Supplementary figures and images for: siRNA Screen Identifies Trafficking Host Factors that Modulate Alphavirus Infection
Source: PLoS Pathog. 2016 Mar 31;12(3):e1005466. doi: 10.1371/journal.ppat.1005466 (PMC4816540; doi:10.1371/journal.ppat.1005466)

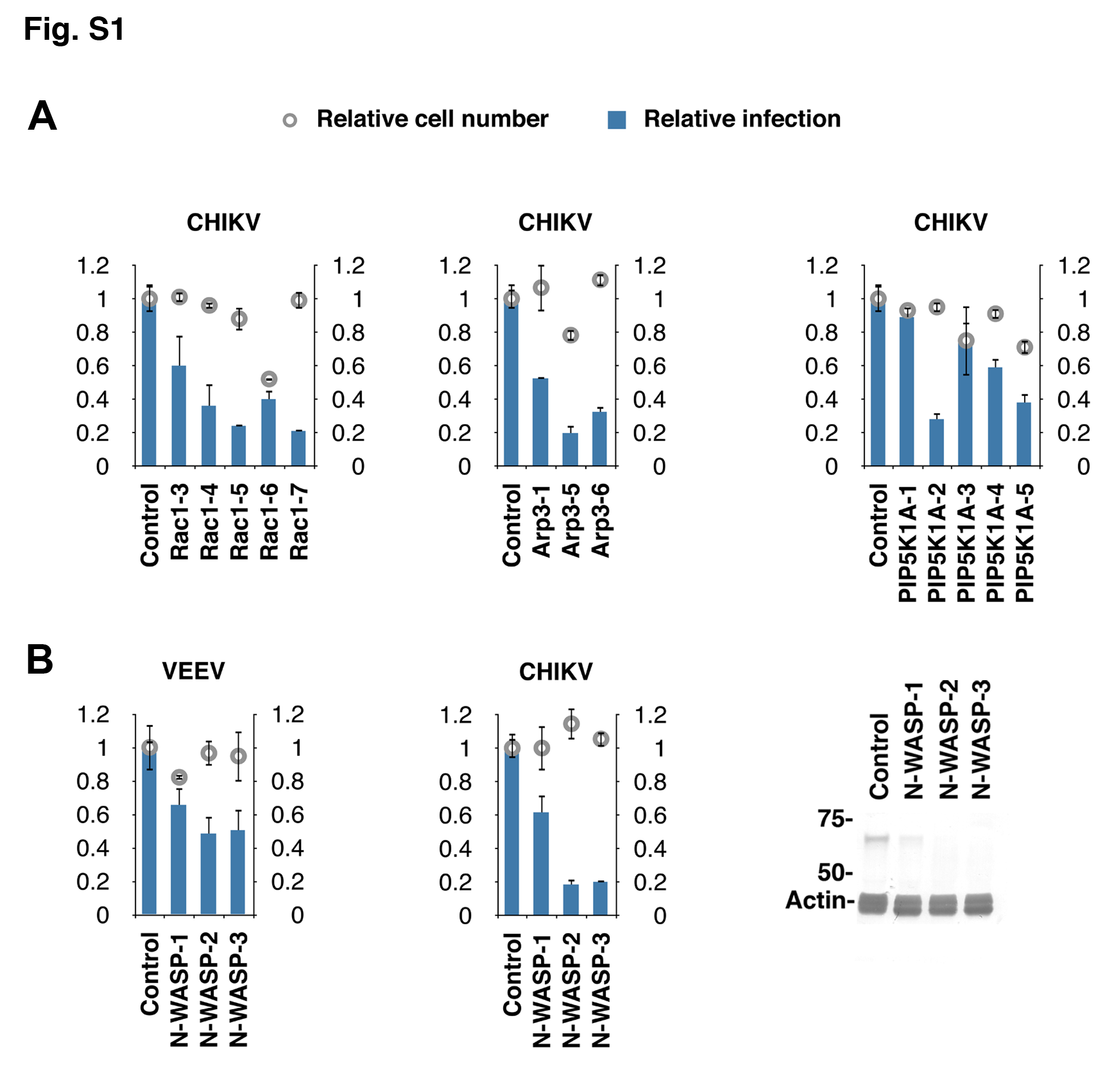

Supplement: S1 Fig — (A) High-content quantitative image-based analysis was used to measure relative infection rates (normalized to control siRNA-treated cells) of CHIKV in HeLa cells pretreated with the indicated siRNAs. Cells were infected for 24 h (CHIKV, MOI = 5), fixed and stained with antibodies against E2. (B) HeLa cells were pretreated with the indicated siRNAs and infected for 20 h with VEEV (MOI = 0.5) or for 24 h with CHIKV (MOI = 5). Cells were fixed, stained, and analyzed as in (A). Protein levels of N-WASP and actin (loading control) following siRNA treatment were determined by immunoblotting (right panel). Values represent the mean ± SD, n = 3. (TIF) [file ppat.1005466.s003.tif]

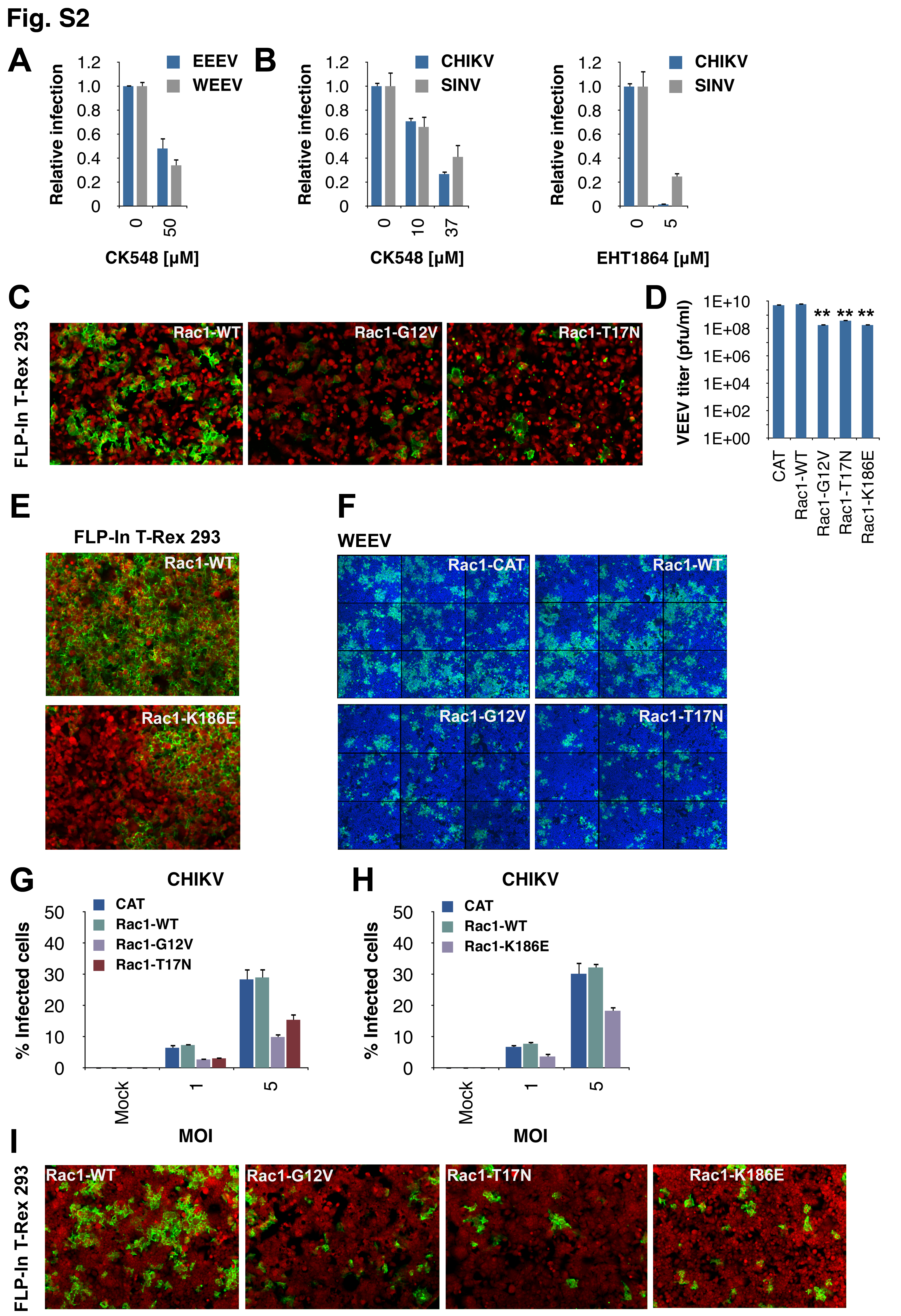

Supplement: S2 Fig — (A) Primary human astrocytes were treated with increasing concentrations of CK548 and subsequently infected with EEEV or WEEV (MOI = 0.005). Cells were fixed in formalin 19 h after infection, stained with virus-specific antibodies, and analyzed using an Opera confocal imager. Results are normalized to DMSO-treated samples. (B) HeLa cells were treated with CK548 or EHT1864 and subsequently infected with CHIKV or SINV (MOI = 5). Cells were fixed 20 h (SINV) or 48 h (CHIKV) later and analyzed as in (A). (C) Representative confocal images of (Fig 2F). VEEV E2 glycoprotein staining is shown in green and nucleus/cytoplasm staining is shown in red. (D) Flp-In T-REx 293 cells pre-induced to express chloramphenicol acetyltransferase (CAT), wild-type Rac1, or variants thereof were infected with VEEV (MOI = 0.1). After 18 h, virus titer in the supernatants was determined by plaque assay. **, p < 0.01, Student's t test (between samples and CAT). (E) Representative confocal images of (Fig 2H). Coloring as in (C). (F) Confocal images of Flp-In T-REx 293 cells that were induced as in (D), inoculated with WEEV (MOI = 0.005), fixed 18 h later, and stained with virus-specific antibodies (green) and nuclear stain (blue). (G, H) High-content quantitative image-based analysis of CHIKV infection rates in Flp-In T-REx 293 cells pre-induced as in (D). Cells were fixed 24 h after virus inoculation and stained with virus-specific antibodies. (I) Representative confocal images of (G, H). CHIKV E2 glycoprotein staining is shown in green and nucleus/cytoplasm staining is shown in red. All values represent the mean ± SD, n = 3. (TIF) [file ppat.1005466.s004.tif]

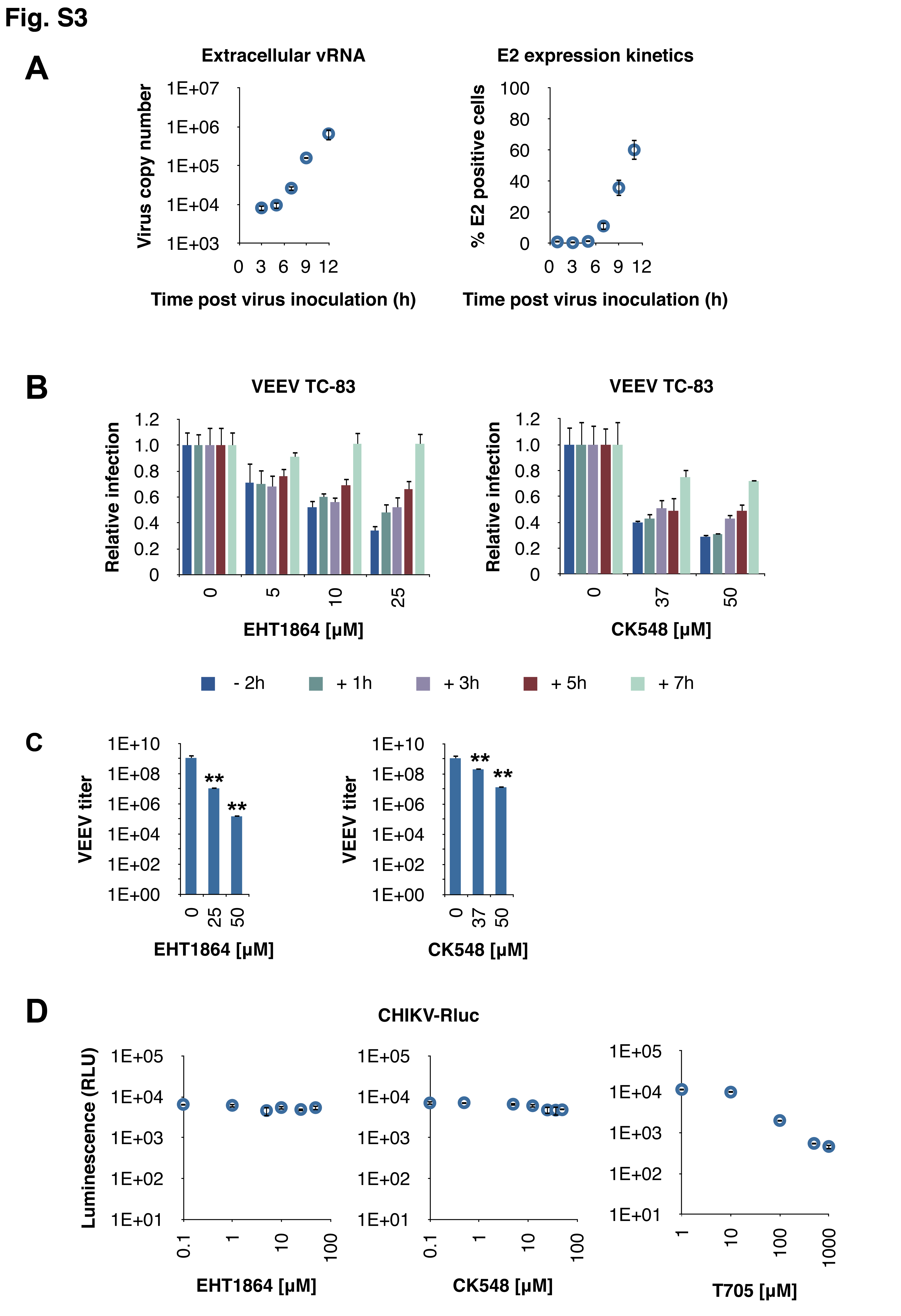

Supplement: S3 Fig — (A) Time course of VEEV TC-83 (MOI = 10) infection in HeLa cells. Media containing extracellular virus were harvested at the indicated time points for qRT-PCR analysis of virion copy number (left panel). Infected cells were fixed, stained with VEEV E2-specific antibody, and analyzed with an Opera confocal reader by high-content quantitative image-based analysis (right panel). (B) High-content quantitative image-based analysis of relative VEEV TC-83 infection rates (normalized to DMSO-treated samples) in time-of-addition experiments. VEEV-infected HeLa cells (MOI = 1) were treated with increasing concentrations of the Rac1 inhibitor EHT1864, or the Arp3 inhibitor CK548 at the indicated time points prior to (-1 h) or after (+1–7 h) virus addition. Cells were fixed 12 h after addition of virus and stained with virus-specific antibodies. Values represent the mean ± SD, n = 3. (C) Plaque assays were used to measure VEEV titer in supernatants of infected HeLa cells treated with the indicated concentrations of the inhibitors. Cells were treated with inhibitors 5 h after inoculation with VEEV (MOI = 0.5), and virus-containing media was harvested for analysis 17 h later. Values represent the mean ± SD, n = 3. **, p < 0.01, Student's t test (between samples and DMSO). (D) BHK-CHIKV-NCT cells expressing a CHIKV replicon with a Renilla luciferase reporter were treated with increasing concentrations of EHT1864, CK548, or T705 (a nucleotide prodrug, positive control). After 48 h, Renilla luciferase (Rluc) activity was determined from the lysates. (TIF) [file ppat.1005466.s005.tif]

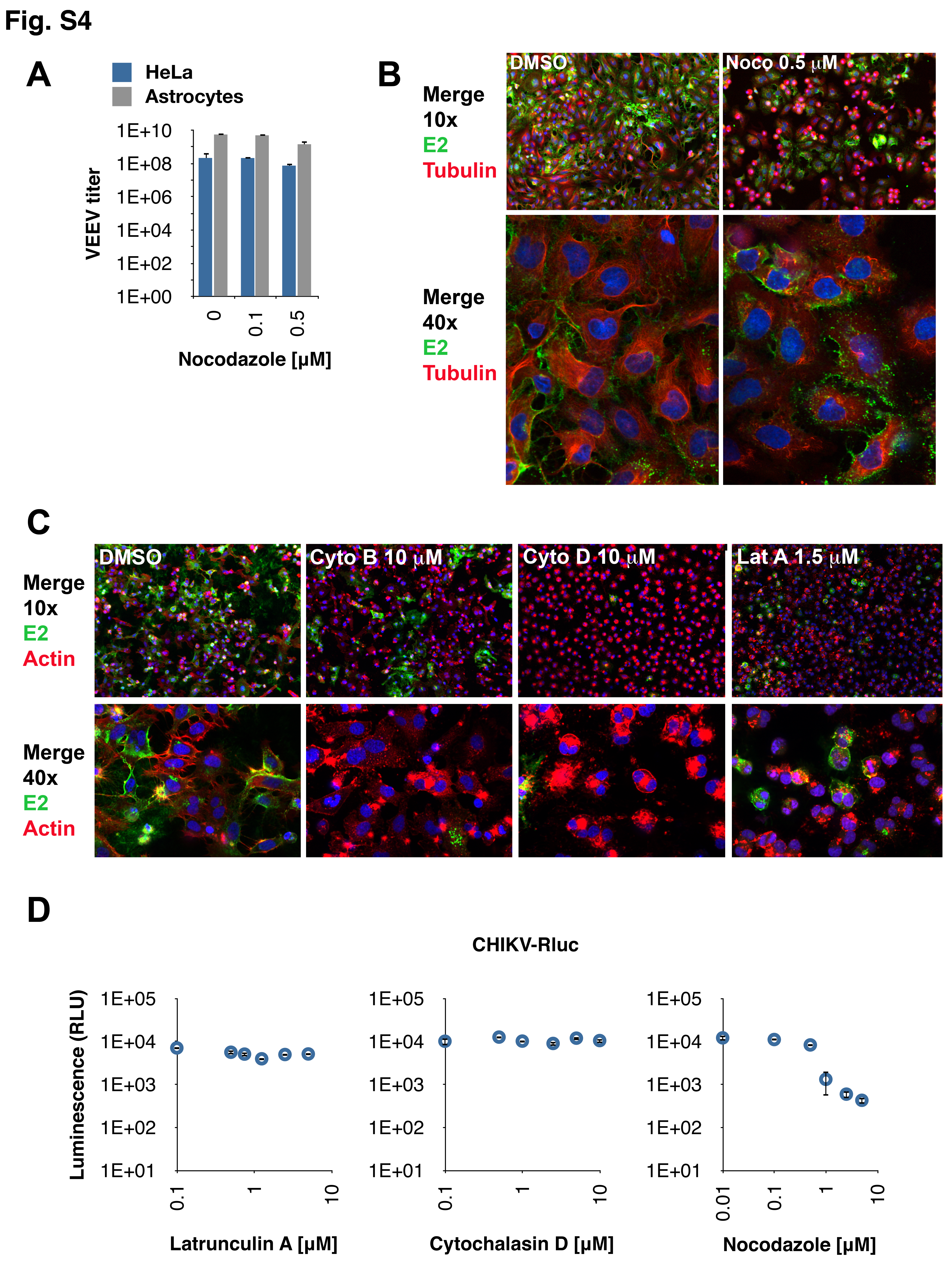

Supplement: S4 Fig — (A) HeLa cells or primary human astrocytes were infected with VEEV (MOI = 0.5) or VEEV TC-83 (MOI = 0.005) for 3 h (HeLa) or 5 h (astrocytes) and then treated with increasing concentrations of nocodazole. After 6 h (astrocytes) or 17 h (HeLa), virus titer in the supernatants was determined by plaque assay. Values represent the mean ± SD, n = 3. (B-C) Representative confocal images of (Fig 4C). VEEV E2 staining is shown in green, nucleus staining is shown in blue, and tubulin (B) or actin (C) staining is shown in red (top panel: magnification: 10x; bottom panel: magnification: 40x). (D) BHK-CHIKV-NCT cells expressing a CHIKV replicon with a Renilla luciferase reporter were treated with increasing concentrations of the indicated inhibitors. After 48 h, Renilla luciferase (Rluc) activity was determined from the lysates. (TIF) [file ppat.1005466.s006.tif]

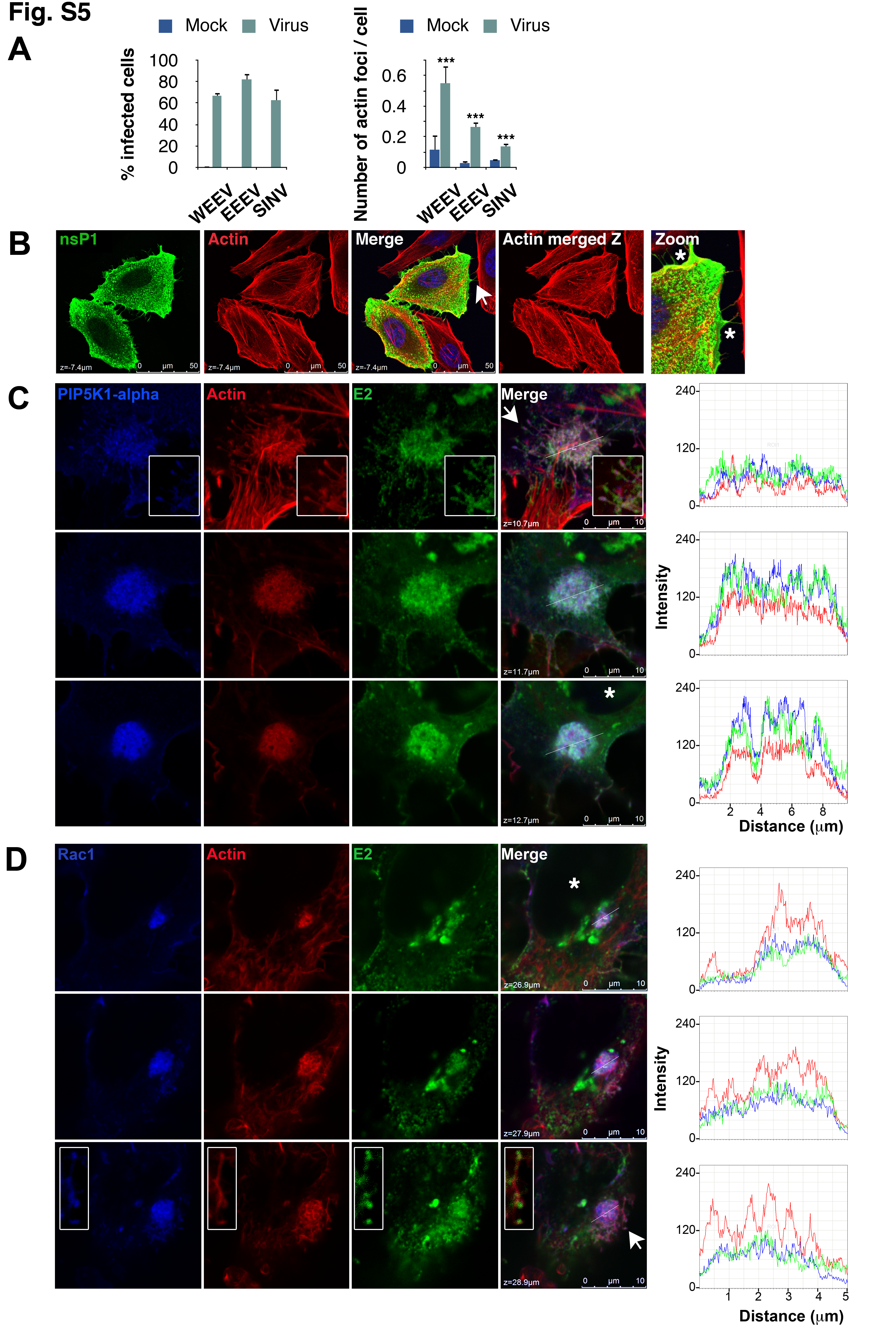

Supplement: S5 Fig — (A) HeLa cells were inoculated with WEEV (MOI = 2), EEEV (MOI = 1), or SINV (MOI = 5), fixed 24 h later, and stained with virus-specific antibodies and fluorescent phalloidin. High-content quantitative image-based analysis was used to measure virus infection rates (left panel) as well as number of actin foci per cell (right panel). ***, p < 0.0001, Student's t test (between samples and mock). (B) HeLa cells were transfected with expression plasmids encoding VEEV nsP1-FLAG. Cells were fixed 24 h later and stained with antibodies against FLAG (green), and fluorescent phalloidin (red). Confocal images of single Z sections and a Z stack image (merged Z sections) are shown of actin staining. Zoom on actin filopodia indicated by a white arrow is shown (right panel). Representative actin filopodia are indicated by asterisks. (C-D) Basal-to-apical confocal section series of VEEV-infected HeLa cells (MOI = 5). Co-localization of HA-tagged PIP5K1-α (C) or Rac1 (D) (blue), actin (red), and VEEV E2 (green), at different Z sections is shown. Insets: zoom on actin filaments indicated by white arrows. Nuclei are indicated by asterisks. Single channel intensities were measured along lines crossing different actin clusters (right panels). VEEV was added to (C) HeLa cells that were reverse-transfected with a plasmid encoding HA-tagged PIP5K1-α or (D) tetracycline-induced T-Rex HeLa cells that expressed Rac1 fused to eGFP. Cells were fixed 20 h later, permeabilized, and stained with VEEV E2-specific antibody, phalloidin, and an antibody against HA (C). (TIF) [file ppat.1005466.s007.tif]

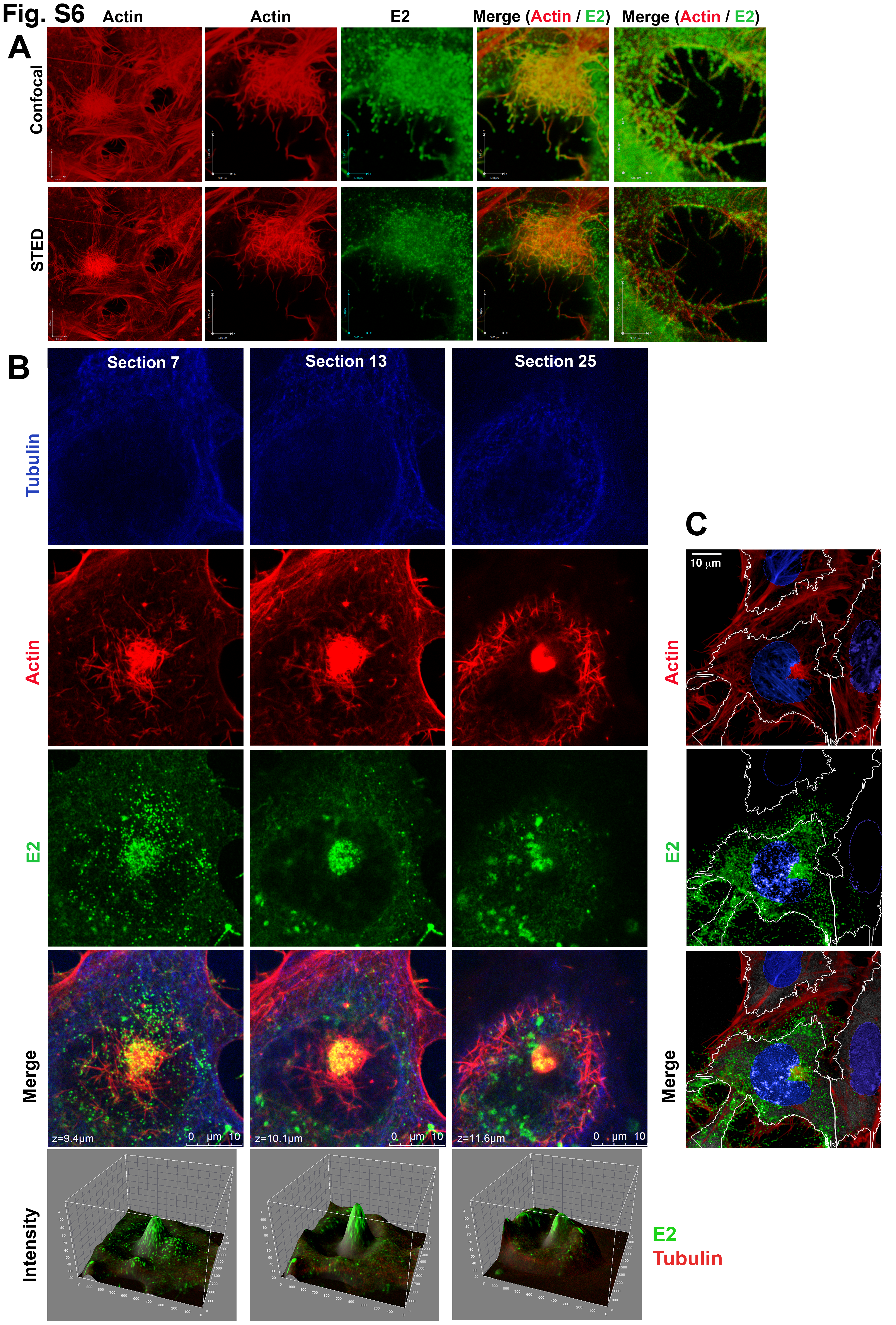

Supplement: S6 Fig — (A) Representative images of VEEV-infected HeLa cells from Fig 6A in confocal and STED microscopy modes. E2 glycoprotein is shown in green and actin in red. (B) Co-localization of tubulin (blue), actin (red), and E2 (green) in a VEEV-infected cell at different Z sections from base (Section 7) to apex (section 25). HeLa cells were infected with VEEV (MOI = 5) for 20 h and stained with antibodies against E2, tubulin, and fluorescent phalloidin. Pixel intensities of tubulin (red) and E2 (green) staining are shown (bottom graphs). (C) Representative images of VEEV-infected HeLa cells (as in B) stained with E2-specific antibodies (green), phalloidin (red) and CellMask (grey in merge). Analysis of cell borders based on CellMask staining was performed within the Columbus programming environment. (TIF) [file ppat.1005466.s008.tif]

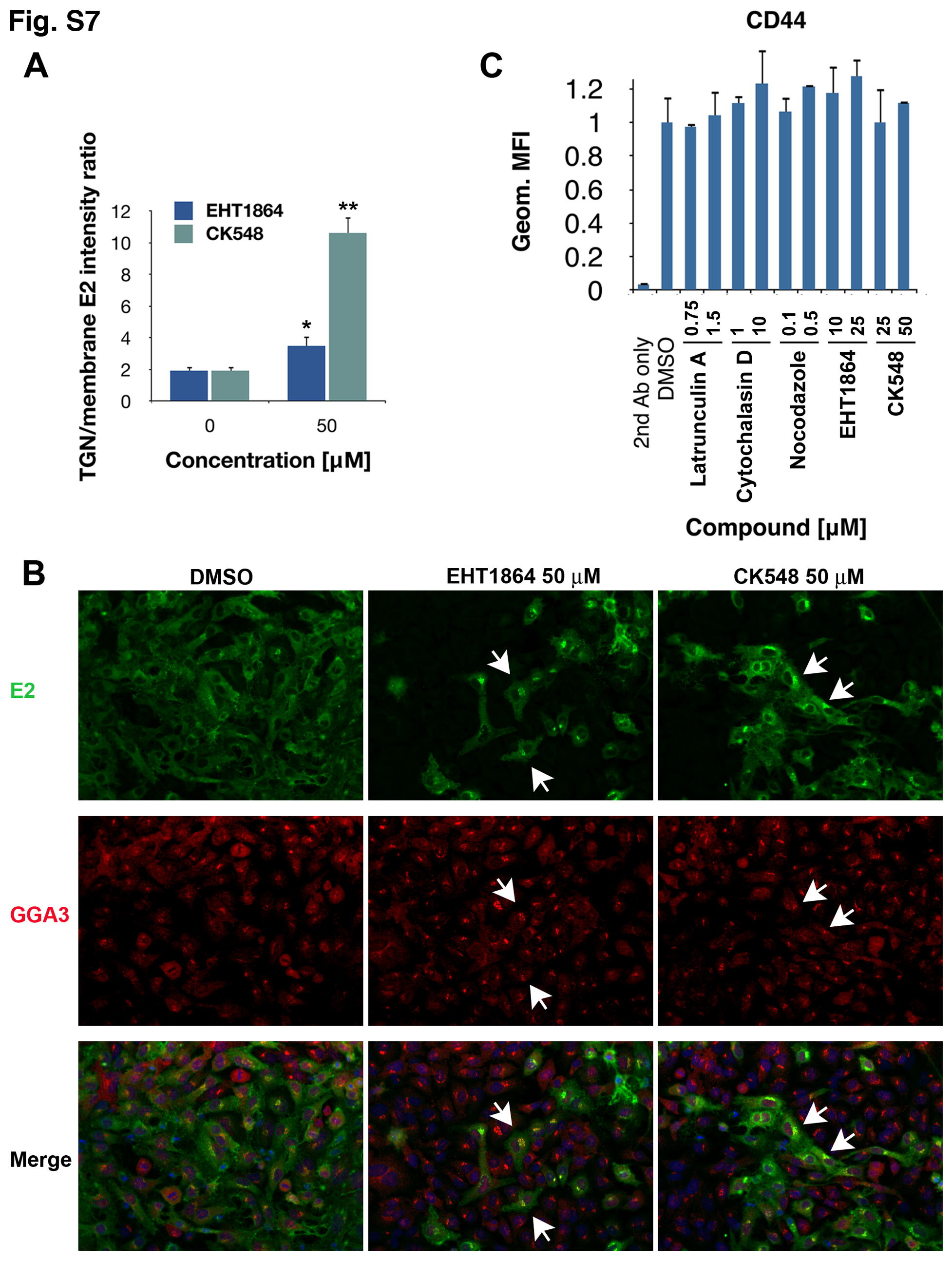

Supplement: S7 Fig — (A) High-content quantitative image-based analysis was used to measure the TGN46-to-plasma membrane E2 staining intensity ratio in VEEV-infected astrocytes. *, p < 0.05, **, p< 0.001, Student's t test (between samples and DMSO). (B) Representative confocal images of HeLa cells treated with DMSO, EHT1864, or CK548 at the indicated concentrations and subsequently infected with VEEV (MOI = 0.5). Cells were fixed and stained with VEEV E2 (green) and GGA3 (red)-specific antibodies and counterstained with Hoechst 3342 (blue) 20 h after infection (magnification: 40x). Representative cells showing co-localization of E2 and GGA3 are indicated with white arrows. (C) Geometrical mean fluorescent intensity of cell-surface CD44 staining in HeLa cells treated with EHT1864, CK548, cytochalasin D, latrunculin A, or nocodazole as measured by flow cytometry. HeLa cells were treated with increasing concentrations of the inhibitors or DMSO (control). Six h later cells were dissociated and stained against CD44 and with a 7-AAD viability dye. (TIF) [file ppat.1005466.s009.tif]

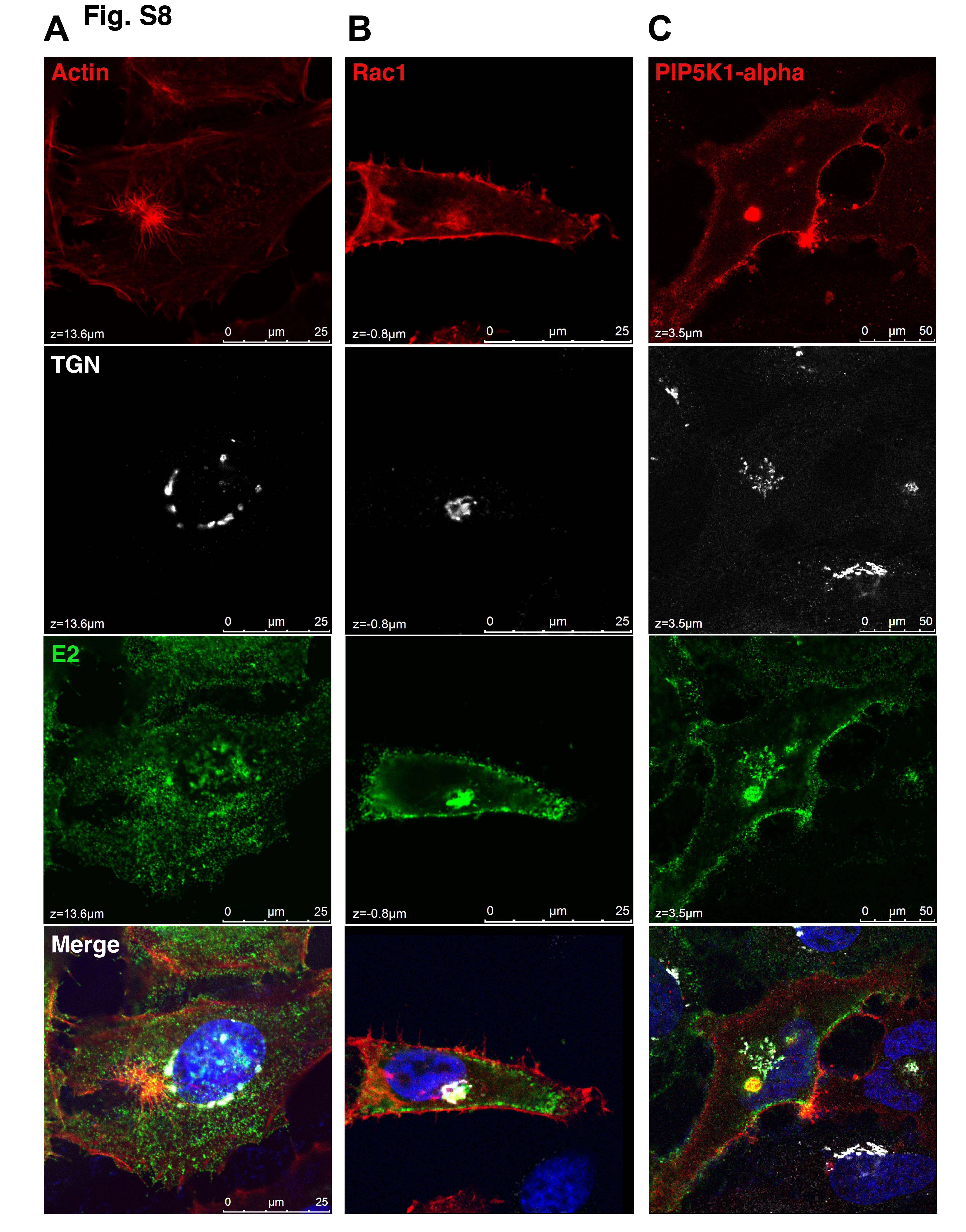

Supplement: S8 Fig — VEEV (MOI = 5) was added to (A) HeLa cells, or (B) tetracycline-induced T-Rex HeLa cells that express Rac1 fused to eGFP or (C) HeLa cells that were reverse-transfected with a plasmid encoding HA-tagged PIP5K1-α. Cells were fixed 20 h later, permeabilized, and stained with VEEV E2- and TGN46-specific antibodies, as well as with phalloidin (A), and an antibody against HA (C). Co-localization of actin (A), Rac1 (B), or PIP5K1-α (C) with TGN46 (white), and VEEV E2 (green), at a single Z section. (TIF) [file ppat.1005466.s010.tif]
